# A Novel Small RNA Promotes Motility and Virulence of Enterohemorrhagic *Escherichia coli* O157:H7 in Response to Ammonium

**DOI:** 10.1128/mBio.03605-20

**Published:** 2021-03-09

**Authors:** Tianyuan Jia, Bin Liu, Huiqian Mu, Chengqian Qian, Lu Wang, Linxing Li, Gege Lu, Wenxuan Zhu, Xi Guo, Bin Yang, Di Huang, Lu Feng, Bin Liu

**Affiliations:** a The Key Laboratory of Molecular Microbiology and Technology, Ministry of Education, Tianjin, People’s Republic of China; b TEDA Institute of Biological Sciences and Biotechnology, Nankai University, TEDA, Tianjin, People’s Republic of China; c Center for Microbial Functional Genomics and Detection Technology, Ministry of Education, Tianjin, People’s Republic of China; d Tianjin Key Laboratory of Microbial Functional Genomics, Tianjin, People’s Republic of China; University of Virginia School of Medicine; Harvard Medical School

**Keywords:** O157:H7, virulence, small RNA, flagella, NtrC

## Abstract

Enterohemorrhagic Escherichia coli serotype O157:H7 (O157) is a critical, foodborne, human intestinal pathogen that causes severe acute hemorrhagic diarrhea, abdominal cramping, and even death. Small RNAs (sRNAs) are noncoding regulatory molecules that sense environmental changes and trigger various virulence-related signaling pathways; however, few such sRNAs have been identified in O157. Here, we report a novel sRNA, EsrF that senses high ammonium concentrations in the colon and enhances O157 pathogenicity by promoting bacterial motility and adhesion to host cells. Specifically, EsrF was found to directly interact with the 5′ untranslated regions of the flagellar biosynthetic gene, *flhB*, mRNA and increase its abundance, thereby upregulating expression of essential flagellar genes, including *flhD*, *flhC*, *fliA*, and *fliC*, leading to elevated O157 motility and virulence. Meanwhile, an infant rabbit model of O157 infection showed that deletion of *esrF* and *flhB* significantly attenuates O157 pathogenicity. Furthermore, NtrC—the response regulator of the NtrC/B two-component system—was found to exert direct, negative regulation of *esrF* expression. Meanwhile, high ammonium concentrations in the colon release the inhibitory effect of NtrC on *esrF*, thereby enhancing its expression and subsequently promoting bacterial colonization in the host colon. Our work reveals a novel, sRNA-centered, virulence-related signaling pathway in O157 that senses high ammonium concentrations. These findings provide novel insights for future research on O157 pathogenesis and targeted treatment strategies.

## Abstract (precis)

The process by which bacteria sense environmental cues to regulate their virulence is complex. Several studies have focused on regulating the expression of the locus of enterocyte effacement pathogenicity island in the typical gut pathogenic bacterium, O157.

## INTRODUCTION

Enterohemorrhagic Escherichia coli (EHEC) serotype O157:H7 (O157) is a foodborne pathogen that causes bloody diarrhea, hemorrhagic colitis, and fatal hemolytic-uremic syndrome in humans (1). O157 usually infects the human colon through contaminated food and water at a low infectious dose (50 CFU) (2). Since no vaccine is available against O157 infections, effective treatments are urgently required to combat these infections (3). O157 typically colonizes the human large intestine (4), and successful O157 colonization is characterized by the formation of attaching and effacing (AE) lesions in the host epithelium (5). These lesions lead to the rearrangement of the actin cytoskeleton and effacement of microvilli, which results in the form of pedestal-like structures and intimate attachment of O157 to the enterocytes (6). The locus of enterocyte effacement (LEE) pathogenicity island mainly encodes genes whose products cause AE lesions (7). The LEE contains five polycistronic operons (LEE1 to LEE5) that express the master LEE regulator, Ler, type III secretion system (T3SS), adhesins (such as intimin and its receptor [Tir]), and other effector proteins (8). In addition to T3SS and effectors encoded by LEE, other adhesins also participate in the initial attachment of bacteria to eukaryotic cells (9).

Flagella are the locomotive organelles important for bacterial pathogenesis and contribute to the initial breakdown and penetration through the mucus layer by bacterial pathogens (10). Moreover, flagella may directly regulate bacterial adhesion and colonization at the sites of infection (11). EHEC flagella directly interact with mucin in the mucus layer (12). O157 flagella also act as adhesins, directly contributing to adhesion to bovine intestinal epithelium in the early stages of colonization (13). Flagella also contribute to the pathogenesis of EHEC O113:H21 infections by promoting the invasion of the intestinal epithelium (14). More than 60 flagellum-related genes have been arranged in hierarchical order in three classes (14). Class 1 contains the master operon, *flhDC*, the expression of which is needed for transcribing class 2 and class 3 operons (15). Class 2 contains eight operons that encode components for constructing the hook-basal body complex, including the sigma factor *fliA*, to directly regulate flagellar genes (16). Class 3 encodes components for filament assembly and motor function, including the flagellin gene, *fliC*. FlhB is a class 2 constituent membrane protein of the basal body required for flagellar appendant synthesis and hook-length control involved in substrate specificity switch (17, 18). Recently, FlhB was found to regulate flagellar gene expression in Listeria monocytogenes (17).

Small RNAs (sRNAs) are regulatory, noncoding, rapid-acting molecules whose synthesis does not require much energy consumption and are involved in many physiological processes of bacteria (19, 20). sRNAs positively or negatively regulate target genes by base pairing with specific mRNAs and often need the sRNA chaperone, Sm-like homohexameric ring protein, Hfq, to function (21, 22). For bacterial pathogens, sRNAs play a vital role in their rapid adaptation to the host environment and regulating virulence gene expression (22). Several virulence-related sRNAs have been identified in O157 (23–26). For example, sRNA, DicF, senses low oxygen concentrations in the environment to induce expression of LEE genes to promote the formation of AE lesions and virulence through direct binding with the transcriptional activator, *pchA* mRNA (23). In addition, several sRNAs (DsrA, Arl, Esr41, Spot42, sRNA56, sRNA103, sRNA350, GlmY, and GlmZ) regulate LEE operons in O157 (26–29). However, the exact mechanisms by which most sRNAs in O157 regulate downstream genes and their roles in bacterial virulence remain unclear.

Bacterial two-component systems (TCSs) sense various microenvironmental cues, which are transferred from the cytoplasmic membrane to the cytoplasm to activate several processes (30). TCSs contain a sensor histidine kinase and its cognate DNA-binding response regulator (RR) (31). The conformational change of the membrane histidine kinase delivers the phosphate to the aspartic residue on the RR to activate a domain that modulates gene expression (32). Previous studies have identified several TCSs that are essential for the virulence of O157 (33). For example, CpxA/CpxR TCS senses the neurotransmitter, serotonin, and downregulates O157 LEE gene expression (33). FusK/R TCS senses fucose produced by the commensal Bacteroides thetaiotaomicron to promote O157 colonization (34). QseE/QseF TCS senses epinephrine and sulfate to regulate the effector protein synthesis, EspFu (35). It is likely that more TCSs are involved in the virulence regulatory network of O157; further investigation is required to confirm and clarify their roles. Here, we aimed to investigate the role of an sRNA, EsrF, and the signaling pathway and the mechanism involved in the motility and virulence of O157.

## RESULTS

### Identification and characterization of EsrF in O157.

By analyzing the transcriptomic data of the O157 strain EDL933 during infection of HeLa cells (36), we predicted one potential sRNA in the intergenic region between *ntrB* (*glnL*) and *glnA* and called it EsrF. The transcriptomic data showed that EsrF expression is significantly upregulated by 4.43-fold when infecting HeLa cells compared to its expression in free cultures in Dulbecco modified Eagle medium (DMEM). We inferred that EsrF is associated with O157 virulence.

**FIG 1:**
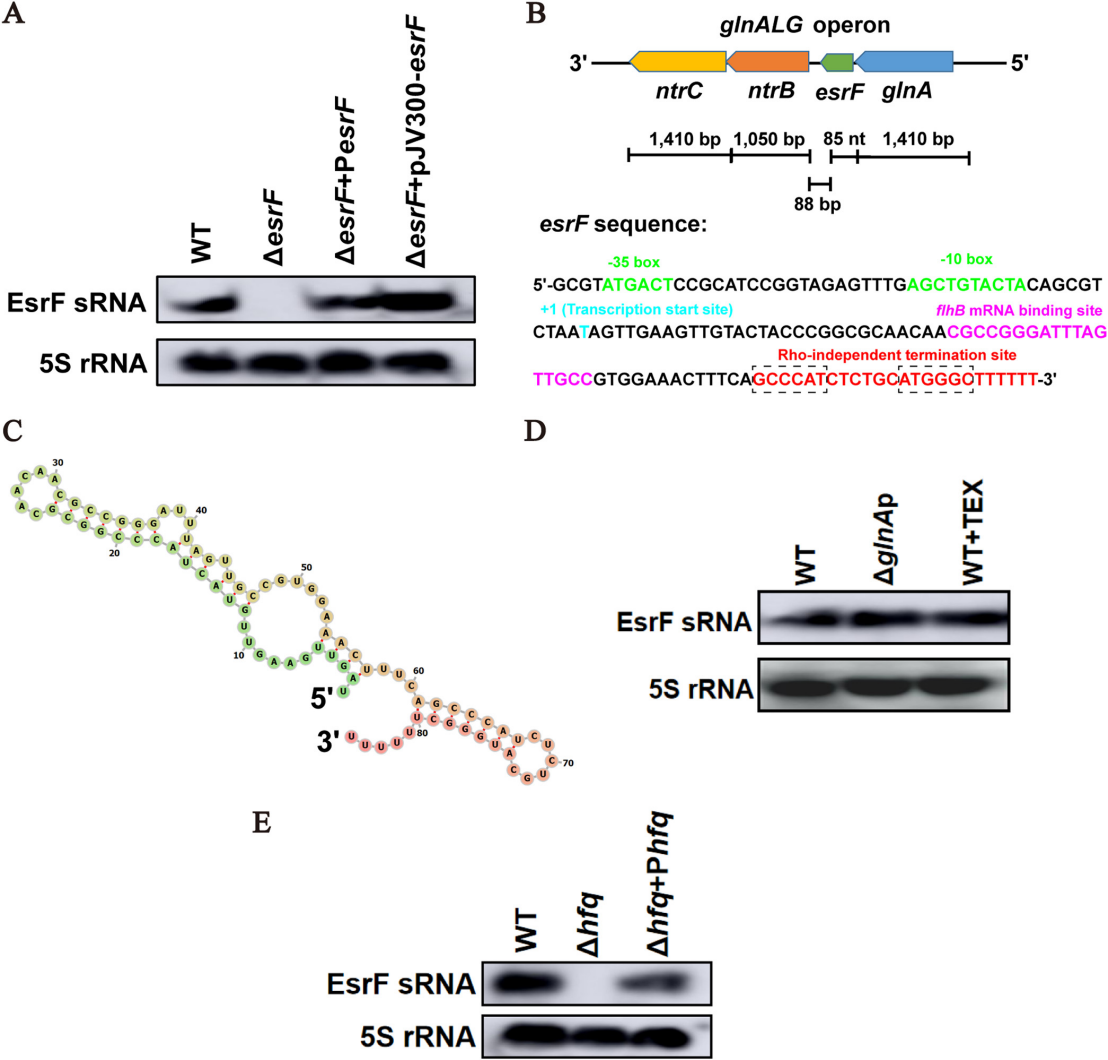
Identification and characterization of EsrF sRNA in O157. (A) Northern blotting performed with a specific probe directed against EsrF sRNA in O157 WT, Δ*esrF*, and Δ*esrF*+P*esrF* strains; 5S rRNA was used as a loading control. *esrF* with its putative promoter was cloned into a high-copy-number plasmid pJV300, and the abundance of EsrF in Δ*esrF*+pJV300-*esrF* was determined by Northern blotting. (B) The position of the *esrF* sequence in the O157 EDL933 genome is shown. The −35 and −10 boxes of the putative promoter region, transcription start site, *flhB* mRNA binding site, and Rho-independent termination site are depicted in green, blue, purple, and red, respectively. The palindromic sequence before poly(T) in Rho-independent termination site are demarcated with a dashed box. (C) Predicted secondary structure of EsrF sRNA determined using RNAfold (Institute for Theoretical Chemistry, University of Vienna [http://rna.tbi.univie.ac.at/]) and depicted as a color gradient from green to red, representing 5′ to 3′ ends of EsrF sRNA, respectively. (D) EsrF abundance in WT, *glnA* promoter mutant (Δ*glnA*p), and WT incubated with 5′ monophosphate-dependent terminator exonuclease (WT+TEX) as determined by Northern blotting. (E) Northern blotting was performed with a specific probe directed against EsrF sRNA in O157 WT, Δ*hfq*, and Δ*hfq*+P*hfq* strains; 5S rRNA was used as a loading control. The data are presented as means ± the SD (*n* = 3; ns, no significance; *, *P* ≤ 0.05; **, *P* ≤ 0.01; ***, *P* ≤ 0.001). All *P* values were calculated using a Student *t* test.

Northern blotting was performed to confirm whether EsrF is an sRNA transcribed in O157 and to determine its size. Briefly, we used an EsrF-specific probe to detect O157 WT (wild type), Δ*esrF* (*esrF* mutant), and Δ*esrF*+P*esrF* (*esrF* complement strain) strains, with 5S rRNA as the internal control. The results revealed an RNA band that was approximately 80 to 90 nucleotides (nt) in length in WT and Δ*esrF*+P*esrF* strains, but not in the Δ*esrF* mutant, indicating that EsrF was transcribed from the reverse strand of the EDL933 genome (Fig. 1A).

We then performed the 5′ and 3′ rapid amplification of cDNA ends (RACE) assay to identify the transcription start and termination sites of EsrF. The results show that EsrF was exactly 85 nt in length, which is consistent with the Northern blotting results. Further, the transcription start site (+1) of EsrF coding sequence (CDS) was mapped to T_4939965_ in the EDL933 genome, while a Rho-independent terminator was mapped to T_4940049_ (Fig. 1B; see also Fig. S1B in the supplemental material). Using RNAfold (37), we also predicted the secondary structure of EsrF to have one hairpin, two bulges, and three internal loops (Fig. 1C).

10.1128/mBio.03605-20.1 FIG S1 EsrF expression, verification, and dependency on Hfq. (A) Transcription of the *esrF-glnA* region as observed in transcriptome data. EsrF appears to be transcribed from its promoter. (B) 5′- and 3′-RACE gel photographs of EsrF cloned into the T vector amplified before sequencing. For 5′-RACE, the size of the product was 130-bp (85-bp *esrF* CDS + 45-bp adapter). For 3′-RACE, the size of product was 250-bp (85-bp *esrF* CDS + 30-bp adapter + 135-bp T vector). (C) Sequencing reads recovered from Hfq UV-induced RNA-protein crosslinking and analysis of cDNA by high-throughput sequencing (CRAC) that map to *esrF* (top) and deletions recovered within sequencing reads (below). The data are presented as means ± the SD (ns, no significance; *, *P ≤ *0.05; **, *P ≤ *0.01; ***, *P ≤ *0.001). All *P* values were calculated using a Student *t* test. Download FIG S1, TIF file, 2.4 MB. Copyright © 2021 Jia et al. 2021 Jia et al. https://creativecommons.org/licenses/by/4.0/ This content is distributed under the terms of the Creative Commons Attribution 4.0 International license.

Moreover, the transcription start site, “T,” of *esrF* is the nucleotide adjacent to the *glnA* CDS stop codon (Fig. 1B). Hence, EsrF is a 3′ untranslated region (UTR) type sRNA that may contain the full *glnA* 3′ UTR. To investigate whether EsrF is independently transcribed or cotranscribed and cleaved from the full-length *glnA* transcript, we performed several analyses. First, the transcriptome data suggest that the putative unique promoter region of *esrF* is located −35 to −10 bp upstream of the *esrF* transcription start site (Fig. 1B; see also Fig. S1A). Second, after deletion of the *glnA* promoter in O157 WT and subsequent Northern blotting, no difference was observed in EsrF abundance between Δ*glnA*p and WT strains (Fig. 1D). Third, considering that cotranscribed sRNAs processed from primary transcripts lead to the conversion of 5′ triphosphorylated RNAs to 5′ monophosphorylated RNAs (38), we treated total RNA samples of O157 WT with the 5′ monophosphate-dependent terminator exonuclease (TEX), which degrades processed transcripts while sparing primary transcripts (39). Northern blotting was subsequently performed, which revealed that the EsrF transcript is resistant to TEX treatment (Fig. 1D). Fourth, we introduced a promoter-less plasmid (pJV300) containing *esrF*, with its predicted promoter region, into the Δ*esrF* mutant, which, according to Northern blotting results, lead to the overexpression of EsrF (Fig. 1A). Taken together, these results indicate that EsrF is an independently transcribed 3′ UTR sRNA, the expression of which is regulated by its promoter and does not require *glnA* expression.

Hfq is a well-known sRNA chaperone that binds to both sRNAs and their target mRNAs to promote their base pairing, while protecting sRNAs from degradation by cellular nucleosidases. The expression of Hfq-binding sRNAs is generally reduced in Gram-negative bacteria *hfq* deletion mutants. We therefore examined the effect of *hfq* deletion on EsrF abundance by performing Northern blotting. Results for which demonstrate the presence of EsrF RNA in WT and Δ*hfq*+P*hfq* strains, which was absent from the Δ*hfq* strain (Fig. 1E), indicating that *hfq* promotes EsrF abundance.

Ultraviolet (UV) cross-linking has been commonly employed to determine whether a specific protein interacts directly with a specific RNA in living cells (40). Specifically, UV-induced RNA-protein cross-linking and high-throughput sequencing has been applied to identify transcriptome-wide targets of Hfq binding in O157 (41). By analyzing this published raw data, we found that Hfq-cross-linked reads cover the entire sequence of *esrF* (see Fig. S1C), suggesting that Hfq may directly bind to the “body” of EsrF sRNA and contribute to its stability.

### EsrF promotes O157 virulence *in vitro* and *in vivo*.

**FIG 2:**
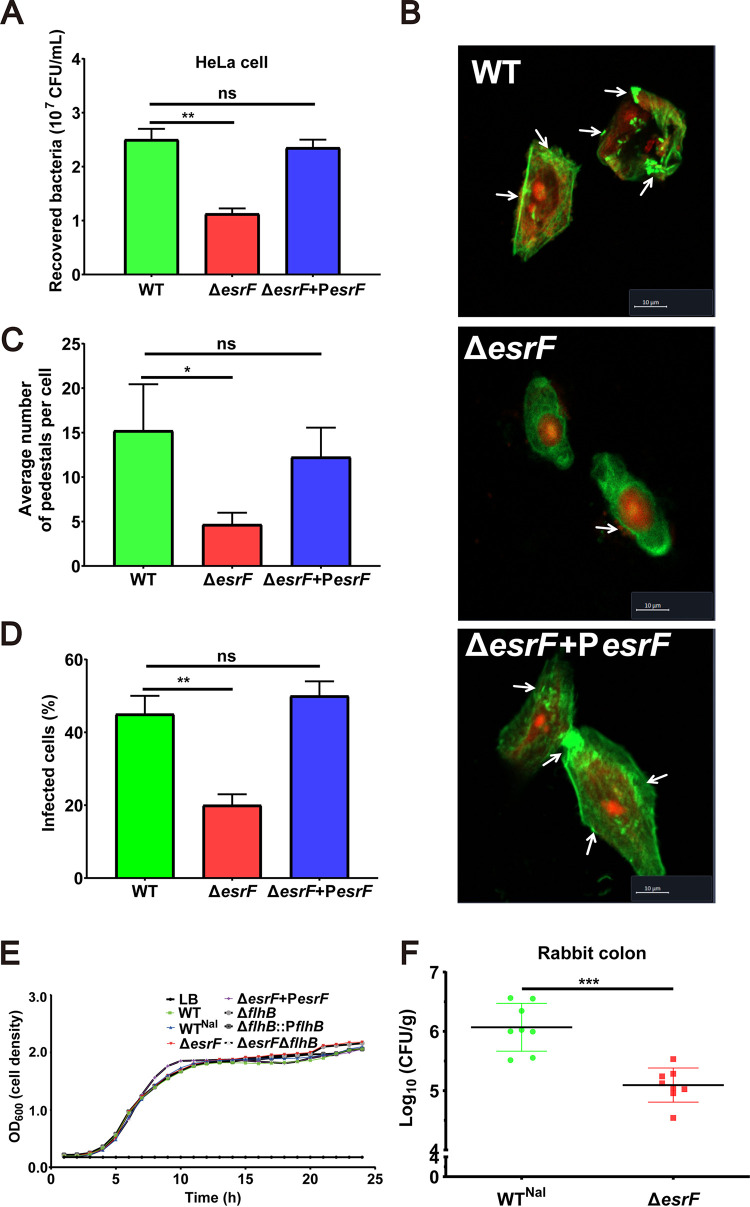
EsrF promoted O157 virulence *in vitro* and *in vivo*. (A) Adhesion of O157 WT, Δ*esrF*, and Δ*esrF*+P*esrF* strains to HeLa cells in DMEM. (B) Quantification of the number of pedestals per infected HeLa cell. (C) Quantification of the proportion of infected HeLa cells. (D) FAS of HeLa cells infected with WT, Δ*esrF*, and Δ*esrF*+P*esrF* strains. DNA was stained with propidium iodide (PI, red), and the HeLa cell actin cytoskeleton was stained with FITC-labeled phalloidin (green). Pedestals were observed as punctate green structures typically associated with bacterial cells. Scale bar, 10 μm. (E) Growth curves for the seven strains (WT, WT^Nal^, Δ*esrF*, Δ*esrF*+Δ*esrF*, Δ*flhB*, Δ*flhB*+Δ*flhB*, and Δ*esrF* Δ*flhB*) used in this study. (F) Colonization capacities of WT and Δ*esrF* strains in rabbit colon. The data are presented as means ± the SD (*n* = 3; ns, no significance; *, *P ≤ *0.05; **, *P ≤ *0.01; ***, *P ≤ *0.001). All *P* values were calculated using a Student *t* test.

To investigate the impact of EsrF sRNA on the adhesion of O157 to host cells, we performed a HeLa cell adhesion assay. To avoid the influence of motility on bacterial adhesion, the bacteria were first centrifuged onto the HeLa cells before infection. The results show that the Δ*esrF* mutant adhered to cells at much lower levels (2.3- and 2.1-fold) than did the WT and Δ*esrF*+P*esrF* strains (Fig. 2A).

Considering that the most important features associated with O157 adhesion to host cells are the AE lesion and pedestal formation (23), fluorescein actin staining (FAS) was performed to observe them in HeLa cells infected by WT, Δ*esrF*, or Δ*esrF*+P*esrF* strains. The results revealed significantly reduced AE lesion formation in HeLa cells infected by the Δ*esrF* mutant (20%) compared to cells infected by WT (45%) or Δ*esrF*+P*esrF* strains (50%), with averages of 15.0, 4.8, and 13.3 pedestals per cell observed in cells infected by WT, Δ*esrF*, and Δ*esrF*+P*esrF* strains, respectively (Fig. 2B, C, and D). Notably, WT, Δ*esrF*, and Δ*esrF*+P*esrF* strains grew at similar rates *in vitro* (Fig. 2E), indicating that the decreased cell adhesion of the Δ*esrF* mutant was not due to slower bacterial growth. Cumulatively, these results suggest that EsrF positively regulates O157 adhesion to host HeLa cells.

To further investigate the effect of EsrF sRNA on O157 pathogenicity *in vivo*, we performed a rabbit colon colonization experiment. Three-day-old New Zealand White infant rabbits were intragastrically inoculated with WT and Δ*esrF* strains, respectively, and the numbers of bacteria recovered from the colon homogenates were determined. The results showed that rabbit colons infected with Δ*esrF* had relatively fewer (10.8-fold) bacteria than the colons infected with the WT strain (Fig. 2F). This result indicated that EsrF sRNA had a positive influence on O157 virulence *in vivo*.

### *flhB* expression is positively regulated by EsrF.

sRNAs influence the expression of their target genes by directly interacting with mRNA molecules (42). We predicted 53 putative target genes of EsrF sRNA using TargetRNA2 (43) (see Table S1A in the supplemental material). Comparative transcriptome sequencing was then performed to detect differences in global gene expression profiles between WT and Δ*esrF* strains, which identified a total of 244 genes (187 downregulated genes and 57 upregulated genes) as differentially transcribed (>2-fold) between the two strains (see Table S1B). Among these differentially transcribed genes, only *flhB* was also predicted by TargetRNA2 as a putative target of EsrF sRNA. FlhB is a flagellar export apparatus membrane protein known to induce effector transport (18); however, more recently, FlhB was also found to regulate other flagellar genes in Listeria monocytogenes (44, 45).

**FIG 3:**
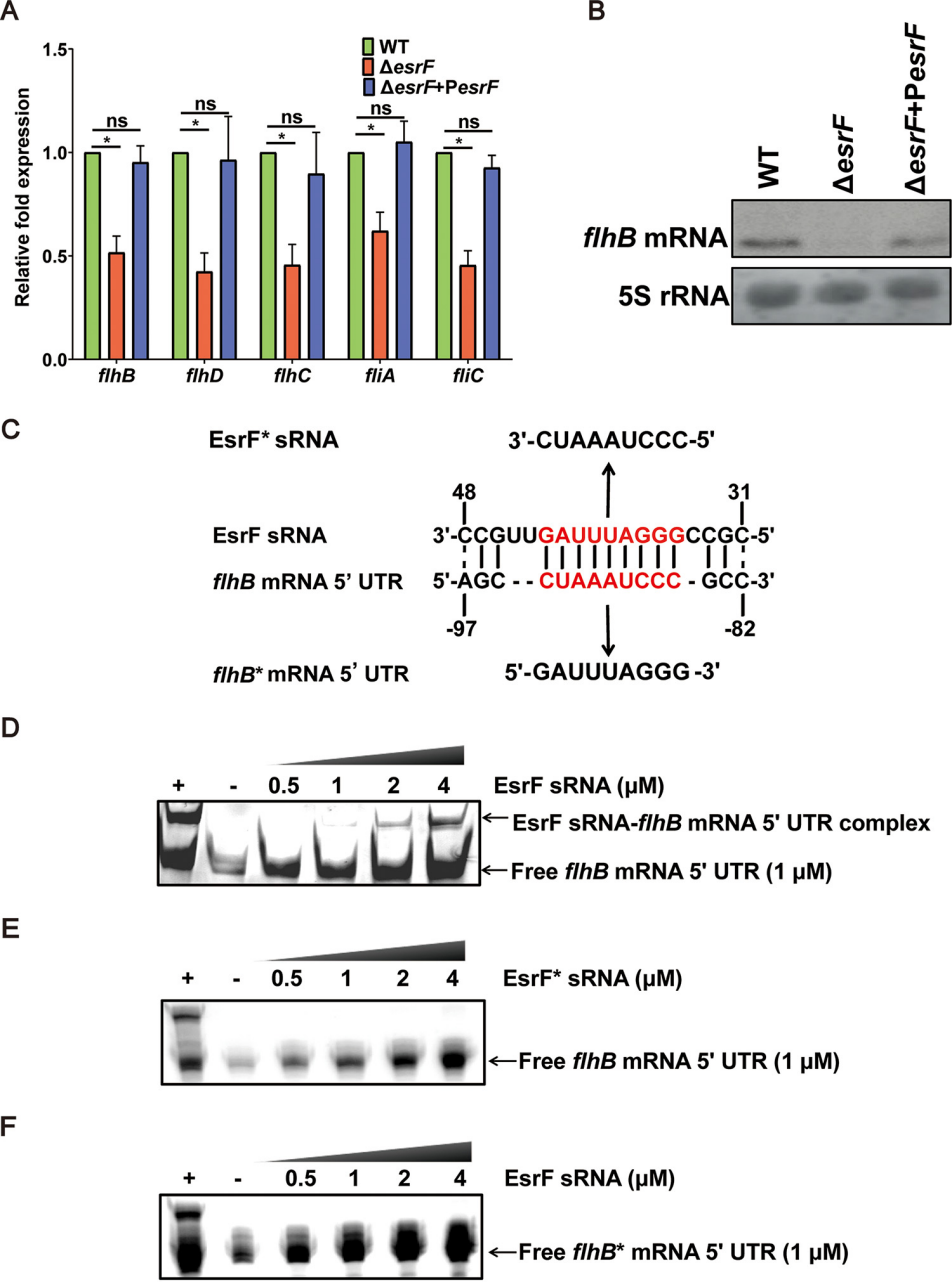
Positive regulation of the downstream gene, *flhB*, by EsrF binding. (A) qRT-PCR of the expression of *flhB*, *flhD*, *flhC*, *fliA*, and *fliC* in WT, Δ*esrF*, and Δ*esrF*+P*esrF* strains. (B) Northern blotting was performed with a specific probe directed against the *flhB* mRNA in O157 WT, Δ*esrF*, and Δ*esrF*+P*esrF* strains; 5S rRNA was used as the loading control. (C) Predicted EsrF sRNA-*flhB* mRNA 5′ UTR base pairing. The lines and dashed lines indicate the predicted base pairs and incomplete base pairs between EsrF and *flhB* RNA 5′ UTR, respectively. The red letters represent the predicted, binding motif for EsrF action. EsrF* sRNA represents EsrF with point mutation with the GGGAUUUAG motif mutated to CCCUAAAUC, while flhB* mRNA 5′ UTR represents *flhB* with point mutations for which the motif CUAAAUCCC was mutated to GAUUUAGGG. (D) RNA-RNA EMSA of EsrF sRNA and *flhB* mRNA 5′ UTR. The EsrF complement strand (+) and yeast RNA (−) were used as positive and negative controls, respectively. (E) RNA-RNA EMSA of EsrF* sRNA and *flhB* mRNA 5′ UTR. (F) RNA-RNA EMSA of EsrF sRNA and flhB* mRNA 5′ UTRs. The data are presented as means ± the SD (*n* = 3; ns, no significance; *, *P ≤ *0.05; **, *P ≤ *0.01; ***, *P ≤ *0.001). All *P* values were calculated using a Student *t* test.

Quantitative reverse transcription-PCR (qRT-PCR) assay results confirmed that *flhB* expression was downregulated by 2.5- and 2.3-fold in the Δ*esrF* mutant, respectively, compared to WT and Δ*esrF*+P*esrF* strains (Fig. 3A). Moreover, *flhB* expression was found to be downregulated by 2.0- and 2.1-fold in Δ*hfq* and Δ*esrF* strains, respectively, compared to the WT (see Fig. S2A). To further confirm the activation of *flhB* by EsrF, we performed Northern blotting to detect *flhB* mRNA levels in WT, Δ*esrF*, and Δ*esrF*+P*esrF* strains. Significantly decreased *flhB* mRNA levels were detected in Δ*esrF* compared to WT, which were restored in the complemented strain (Fig. 3B; see also Fig. S2B). The 18-nt interaction region between EsrF sRNA (CCGUUGAUUUAGGGCCGC) and *flhB* mRNA (AGCCUAAAUCCCGCC) 5′ UTRs was predicted by TargetRNA2 (Fig. 3C).

10.1128/mBio.03605-20.2 FIG S2 EsrF positively regulates *flhB* transcription. (A) qRT-PCR quantification of flhB expression in WT, Δ*esrF*, Δ*esrF*+P*esrF*, Δ*hfq*, and Δ*hfq*+P*hfq* strains. (B) Northern blotting quantification of *flhB* mRNA in WT, Δ*esrF*, and Δ*esrF*+P*esrF* strains. (C) Quantification of shifted *flhB* mRNA in RNA-RNA EMSA reactions. The data are presented as means ± the SD (ns, no significance; *, *P* ≤ 0.05; **, *P* ≤ 0.01; ***, *P* ≤ 0.001). All *P* values were calculated using a Student *t* test. Download FIG S2, TIF file, 1.4 MB. Copyright © 2021 Jia et al. 2021 Jia et al. https://creativecommons.org/licenses/by/4.0/ This content is distributed under the terms of the Creative Commons Attribution 4.0 International license.

10.1128/mBio.03605-20.8 TABLE S1 (A) TargetRNA2 used to predict 53 putative target genes of EsrF. (B) Differently expressed genes of the O157 Δ*esrF* transcriptome. Download Table S1, DOCX file, 0.05 MB. Copyright © 2021 Jia et al. 2021 Jia et al. https://creativecommons.org/licenses/by/4.0/ This content is distributed under the terms of the Creative Commons Attribution 4.0 International license.

RNA-RNA electrophoretic mobility shift assay (REMSA) was performed to verify the direct interaction between EsrF sRNA and *flhB* mRNA *in vitro*. EsrF, the EsrF complementary strand, and *flhB* mRNA were transcribed *in vitro* and purified. The REMSA reaction was performed with different concentrations of EsrF sRNA (0.5, 1, 2, and 4 μM) and a fixed amount of *flhB* mRNA (1 μM). EsrF sRNA-*flhB* mRNA complex bands were formed by both EsrF sRNA and *flhB* mRNA in 8% native polyacrylamide gel electrophoresis (PAGE), and the band intensity increased as the EsrF concentration increased from 0.5 to 4 μM (Fig. 3D; see also Fig. S2C). The REMSA verified the direct interaction between EsrF sRNA and *flhB* mRNA *in vitro* and suggested that EsrF sRNA directly upregulated *flhB* expression.

The 9-nucleotide crucial motif (GGGAUUUAG) for EsrF sRNA action was predicted by TargetRNA2 (Fig. 3C). To determine whether this motif represents the key sequence bound to *flhB* mRNA, we performed REMSA under the same reaction conditions, using a mutated EsrF (EsrF*) sRNA (the motif GGGAUUUAG was mutated to CUCCCTAAA) generated by *in vitro* transcription with *flhB* mRNA. We also performed REMSA using a mutated *flhB* (flhB*) mRNA (the motif CUAAAUCCC was mutated to GAUUUAGGG) with EsrF sRNA. The results showed no interaction between the EsrF* sRNA and *flhB* mRNA, or between EsrF sRNA and flhB* mRNA (Fig. 3E and F), indicating that this motif is vital to the ability of EsrF sRNA to bind to *flhB* mRNA.

### EsrF and *flhB* promote O157 motility.

**FIG 4:**
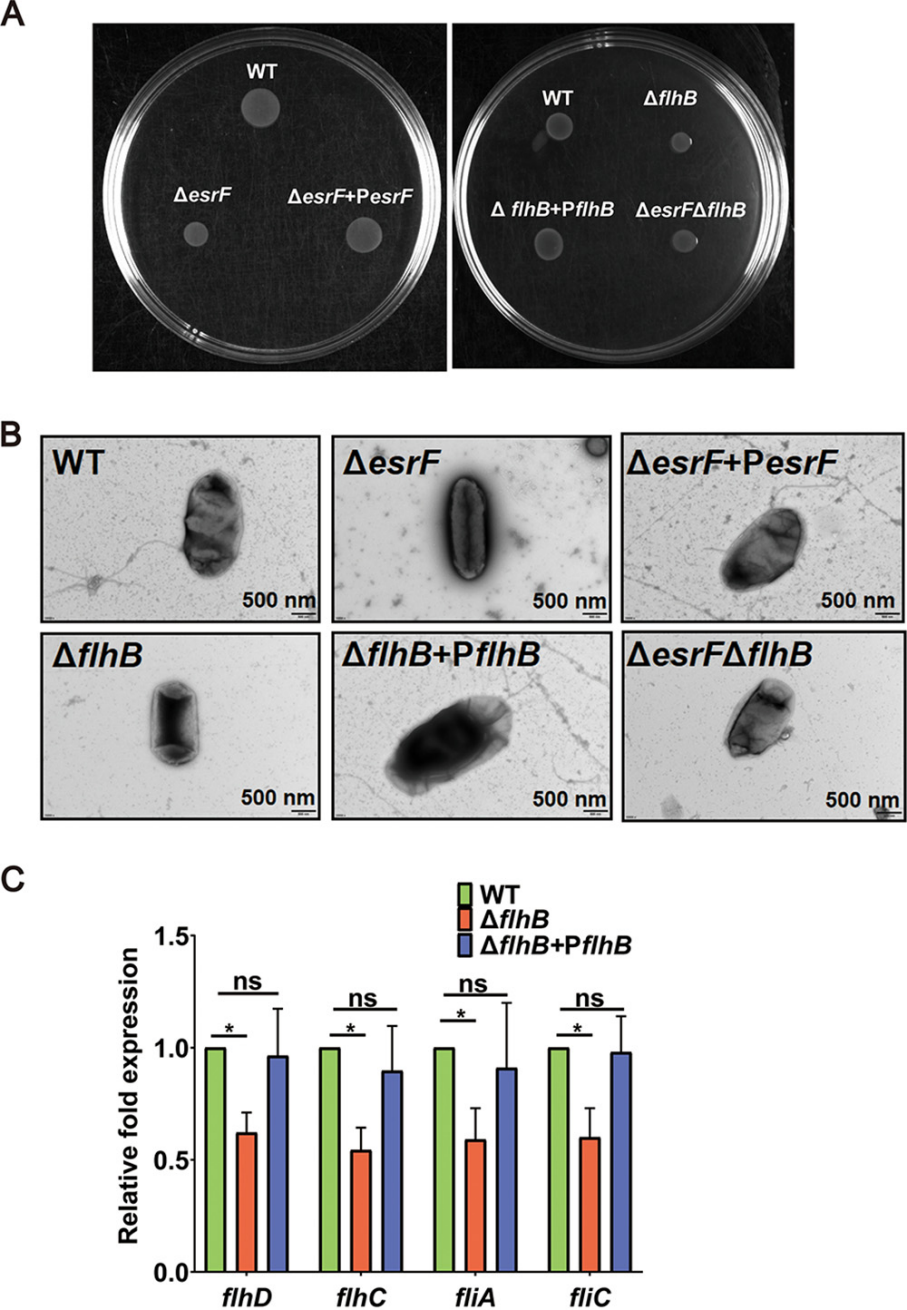
EsrF and *flhB* promotes O157 motility. (A) Representative images of swimming motility of O157 WT, Δ*esrF*, Δ*esrF*+P*esrF*, Δ*flhB*, Δ*flhB*+P*flhB*, and Δ*esrF* Δ*flhB* strains. All strains were observed after 12 h of growth in semisolid LB medium at 25°C. (B) Representative TEM images of O157 WT, Δ*esrF*, Δ*esrF*+P*esrF*, Δ*flhB*, Δ*flhB*+P*flhB*, and Δ*esrF* Δ*flhB* strains. Scale bar, 500 nm. (C) qRT-PCR of the expression of *flhD*, *flhC*, *fliA*, and *fliC* in WT, Δ*flhB*, and Δ*flhB*+P*flhB* strains. The data are presented as means ± the SD (*n* = 3; ns, no significance; *, *P ≤ *0.05; **, *P ≤ *0.01; ***, *P ≤ *0.001). All *P* values were calculated using a Student *t* test.

Considering that flagella are directly related to bacterial motility (11), a swimming motility assay was performed to determine whether *esrF* or *flhB* influence O157 motility. The growth radius was found to decrease significantly in the Δ*esrF* and Δ*flhB* mutants compared to the WT and the corresponding complement strains (Fig. 4A; see also Fig. S3A). Growth curve analysis further demonstrated that WT, Δ*esrF*, and Δ*flhB* strains and the corresponding complement strains grew at similar rates in Luria-Bertani (LB) medium (Fig. 2E), indicating that the decreased motility in Δ*esrF* and Δ*flhB* strains was not due to slower growth. Together, these results indicate that both *esrF* and *flhB* enhance O157 motility.

10.1128/mBio.03605-20.3 FIG S3 EsrF and *flhB* regulate O157 motility and flagella synthesis. (A) Growth radii of WT, Δ*esrF*, Δ*esrF*+P*esrF*, Δ*flhB*, Δ*flhB*+P*flhB*, and Δ*esrF* Δ*flhB* strains in motility assays. (B) Percentages of flagella numbers/20 cells of WT, Δ*esrF*, Δ*esrF*+P*esrF*, Δ*flhB*, Δ*flhB*+P*flhB*, and Δ*esrF* Δ*flhB* strains. The data are presented as means ± the SD (ns, no significance; *, *P ≤ *0.05; **, *P ≤ *0.01; ***, *P ≤ *0.001). All *P* values were calculated using a Student *t* test. Download FIG S3, TIF file, 0.9 MB. Copyright © 2021 Jia et al. 2021 Jia et al. https://creativecommons.org/licenses/by/4.0/ This content is distributed under the terms of the Creative Commons Attribution 4.0 International license.

We further investigated whether flagellar synthesis was inhibited in Δ*esrF* and Δ*flhB* strains by transmission electron microscopy (TEM). The results showed that approximately 75% of the WT and complemented cells possessed flagella, whereas approximately 85 and 90% of Δ*esrF* and Δ*flhB* cells were aflagellate (Fig. 4B; see also Fig. S3B). These results indicated that *esrF* and *flhB* promoted flagellar synthesis, which resulted in enhanced bacterial motility.

Although FlhB is generally regarded as a class 2 flagellar biosynthetic protein (46), recently *flhB* was reported to regulate the expression of several flagellar genes, including *flaA* (O157 *fliC* homolog), *fliY*, *fliS*, *motA*, *lmo0695*, and *lmo0698*, in Listeria monocytogenes (17). Hence, we performed qRT-PCR to determine whether *flhB* regulates the expression of flagellar genes in O157. The expression of *flhD*, *flhC*, *fliA*, and *fliC* was significantly downregulated in the Δ*flhB* strain compared to WT and Δ*flhB*+P*flhB* strains (Fig. 4C). Furthermore, the expression of *flhB*, *flhD*, *flhC*, *fliA*, and *fliC* was also significantly downregulated in the Δ*esrF* mutant compared to WT and Δ*esrF*+P*esrF* strains (Fig. 3A). These results indicate that EsrF sRNA promotes *flhB*-mediated expression of flagellar genes in O157.

To determine whether EsrF influences the motility of O157 directly by regulating *flhB* expression, we constructed an Δ*esrF* Δ*flhB* double mutant. Swimming motility and TEM results showed that the growth radius and flagellar synthesis decreased to similar levels in the Δ*flhB* and Δ*esrF* Δ*flhB* mutants compared to those of the WT strain (Fig. 4A and B). These data indicate that *esrF* contributed to the motility of O157 by directly inducing the expression of *flhB* (Fig. 3 and 4).

### *flhB* promotes O157 virulence *in vitro* and *in vivo*.

**FIG 5:**
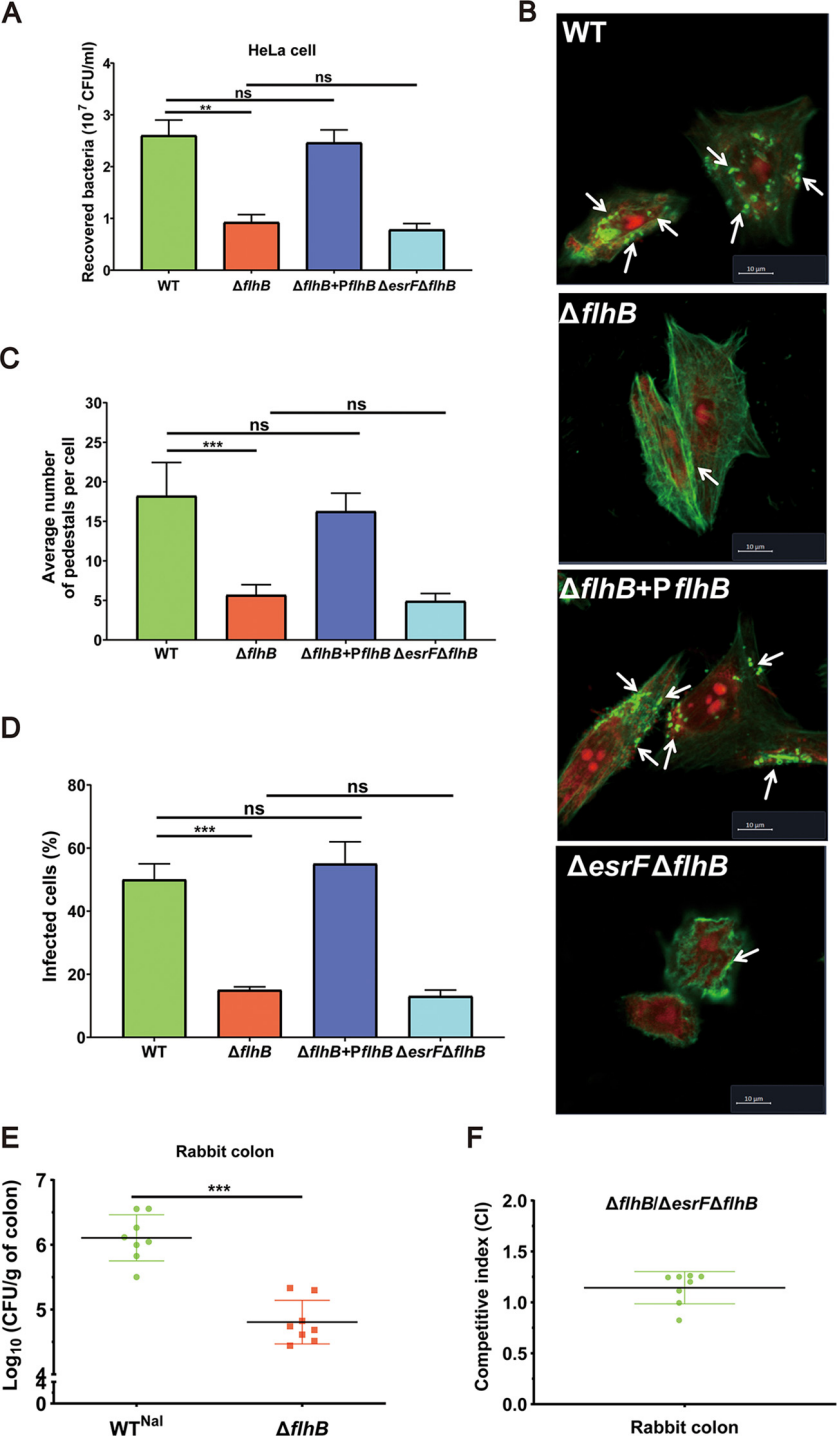
*flhB* promotes O157 virulence *in vitro* and *in vivo*. (A) HeLa cell adhesion of WT, Δ*flhB*, Δ*flhB*+P*flhB*, and Δ*esrF* Δ*flhB* strains at an MOI of 10. (B) FAS of HeLa cells infected with WT, Δ*flhB*, Δ*flhB*+P*flhB*, and Δ*esrF* Δ*flhB* strains. DNA was stained with propidium iodide (PI, red), and the HeLa cell actin cytoskeleton was stained with FITC-labeled phalloidin (green). Pedestals were observed as punctate green structures typically associated with bacterial cells. Scale bar, 10 μm. (C) Quantification of pedestals on HeLa cells infected with WT, Δ*flhB*, Δ*flhB*+P*flhB*, and Δ*esrF* Δ*flhB* strains. (D) Quantification of the proportion of infected HeLa cells. (E) Colonization of rabbit colon by WT and Δ*flhB* strains. (F) The competitive colonization index of Δ*flhB* versus Δ*esrF* Δ*flhB* strains in rabbit colon. The data are presented as means ± the SD (*n* = 3; ns, no significance; *, *P ≤ *0.05; **, *P ≤ *0.01; ***, *P ≤ *0.001). All *P* values were calculated using a Student *t* test.

The effect of *flhB* on O157 virulence was investigated using the HeLa cell adhesion assay and rabbit colon colonization experiments. The results showed that Δ*flhB* cell adhesion decreased significantly compared with that of WT (2.9-fold) and Δ*flhB*+P*flhB* (2.7-fold) strains (Fig. 5A). Moreover, the FAS results revealed significantly reduced AE lesion formation in HeLa cells infected with the Δ*flhB* mutant (15%) compared to WT (50%) and Δ*flhB*+P*flhB* (55%) strains, with averages of 18.3, 5.2, and 15.3 pedestals on each cell infected by WT, Δ*flhB*, and Δ*flhB*+P*flhB* strains, respectively (Fig. 5B to D). Notably, WT, Δ*flhB*, and Δ*flhB*+P*flhB* strains grew at similar rates *in vitro* (Fig. 2E), indicating that the decreased cell adhesion of Δ*flhB* was not due to slower bacterial growth. In addition, an infant rabbit model of O157 infection revealed that significantly fewer bacteria were recovered from rabbit colons infected with the Δ*flhB* mutant (16.7-fold) than with the WT strain (Fig. 5E), thereby validating the inference exerting by *flhB* on O157 virulence *in vitro* and *in vivo*.

We next sought to determine whether EsrF directly influences O157 virulence by regulating *flhB* expression. HeLa cell adhesion and FAS results showed no further significant decrease in cell adhesion or pedestal formation for the Δ*esrF* Δ*flhB* strain compared to the Δ*flhB* mutant (Fig. 5A to D). Moreover, competitive infection assays in rabbits using Δ*flhB* and Δ*esrF* Δ*flhB* mutants revealed that the competitive index (CI) for Δ*esrF* Δ*flhB* versus Δ*flhB* strains in the colonization of rabbit colon was 1.09, indicating a similar colonization ability (Fig. 5F). Together, these data indicate that *esrF* contributes to O157 virulence by directly inducing the expression of *flhB* (Fig. 2 and 5).

### NtrC negatively regulates *esrF* expression.

**FIG 6:**
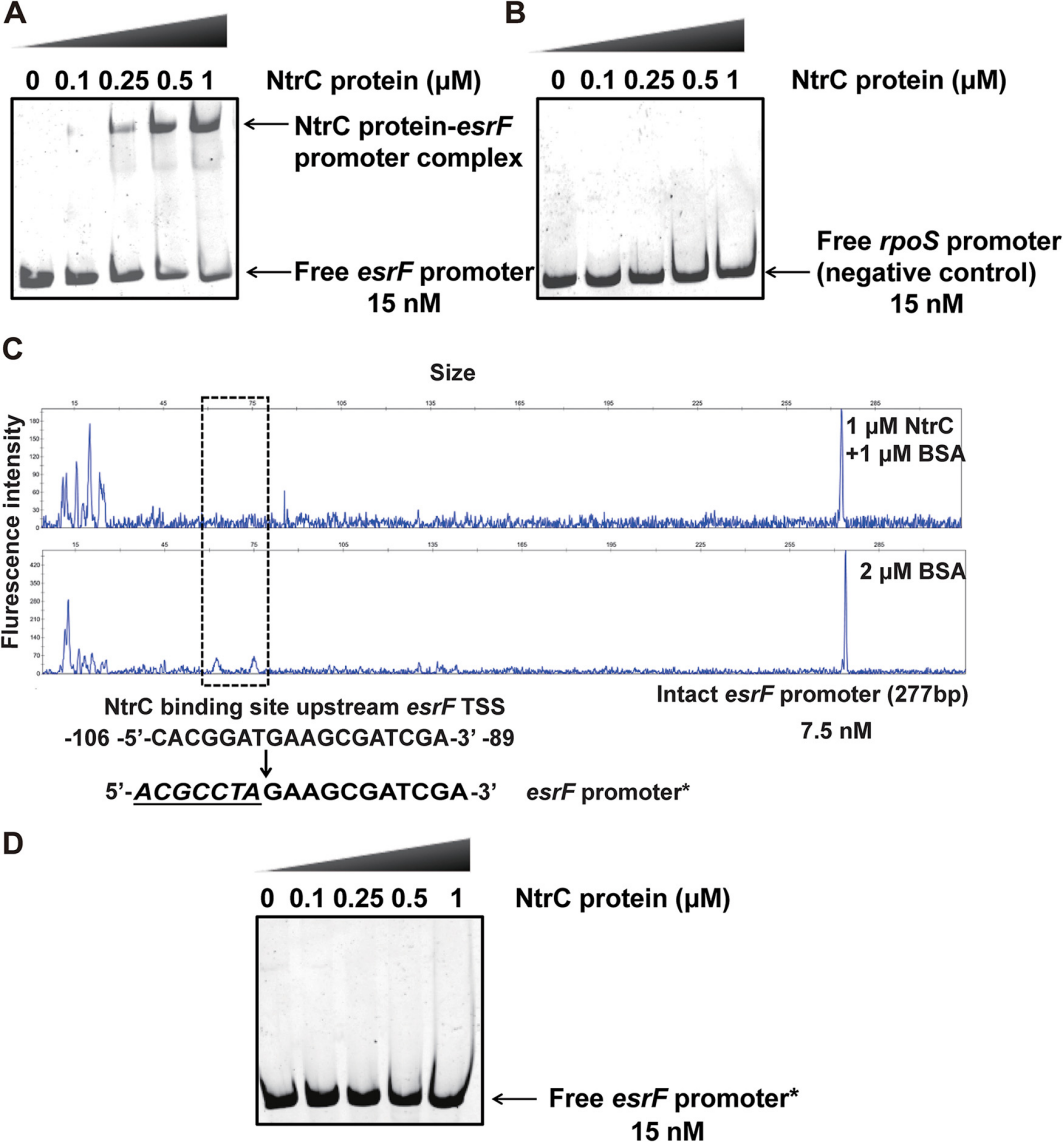
NtrC regulates EsrF by binding to its promoter. (A) EMSAs were performed with purified NtrC protein and *esrF* promoter. (B) EMSAs were performed with purified NtrC protein and *rpoS* promoter, used as the negative control. (C) NtrC protein bound to the motif CACGGATGAAGCGATCGA in the *esrF* promoter region. Electropherograms showed the protection pattern of the *esrF* promoter region after digestion with DNase I after incubation in the absence or presence of 1 μM NtrC protein. (D) EMSAs of NtrC protein with the modified *esrF* promoter region (the motif CACGGATGAAGCGATCGA mutated to *ACGCCTA*GAAGCGATCGA). The data are presented as means ± the SD (*n* = 3; ns, no significance; *, *P ≤ *0.05; **, *P ≤ *0.01; ***, *P ≤ *0.001). All *P* values were calculated using a Student *t* test.

The *esrF* gene sequence is located within the *glnALG* operon of the O157 genome; the expressions of all genes within this operon are regulated by NtrC (47). NtrC is the RR of the NtrC/B TCS, which regulates ∼100 nitrogen (N) starvation genes to balance bacterial N metabolism (47). According to the reported NtrC binding sites in the *glnA* 5′ UTR (NR1 to NR5) (48), we searched the *esrF* upstream region and found one 13-bp potential NtrC binding site (5′-TCACGGATGAAGC-3′; positions −107 to −95 from the *esrF* transcriptional start site [TSS]; see Fig. S4A). The NtrC-His_6_ protein was subsequently purified and used in a protein-DNA promoter EMSA, along with an amplified DNA fragment of the *esrF* promoter region to verify whether the phosphorylated form of NtrC protein (NtrC-P) directly binds to the *esrF* promoter. Slowly migrating bands were observed for the *esrF* promoter with increasing NtrC protein concentrations (Fig. 6A), and the intensities of these bands gradually strengthened as the concentration of NtrC protein increased from 0.25 to 1 μM. Meanwhile, no migrating bands were observed for the negative-control *rpoS* promoter with increasing NtrC protein concentrations (Fig. 6B), thus verifying the direct binding between the NtrC protein to the *esrF* promoter *in vitro*.

10.1128/mBio.03605-20.4 FIG S4 The upstream gene *ntrC* negatively regulates EsrF expression in high ammonium environment. (A) The five previously known NtrC binding sites and potential NtrC binding sites located upstream of *esrF* were aligned using the MUltiple Sequence Comparison by Log-Expectation (MUCLE) program in Unipro Ugene software, with the “gCaC-at+aTggtGc” consensus sequence. (B) Quantification of EsrF abundance in WT and Δ*ntrC* after treatment with 3 mM (low ammonium) and 24 mM (high ammonium) NH_4_Cl. (C) qRT-PCR performed to detect *gfp* expression to represent the *esrF*p and *flhB*p expression of the Δ*ntrC* mutant in the colon *in vivo* and LB medium-cultured samples. *rfp* was used as the internal control. The data are presented as means ± the SD (ns, no significance; *, *P ≤ *0.05; **, *P ≤ *0.01; ***, *P ≤ *0.001). All *P* values were calculated using a Student *t* test. Download FIG S4, TIF file, 2.0 MB. Copyright © 2021 Jia et al. 2021 Jia et al. https://creativecommons.org/licenses/by/4.0/ This content is distributed under the terms of the Creative Commons Attribution 4.0 International license.

Using the dye-based DNase I footprinting assay, we identified a specific NtrC-bound sequence containing an 18-bp motif (5′-CACGGATGAAGCGATCGA-3′; positions −106 to −89 from the *esrF* TSS; Fig. 6C). This motif contains the 13-bp predicted NtrC binding site described above. To determine whether the motif was necessary for binding to NtrC, we performed EMSAs using a mutated *esrF* promoter (the motif CACGGATGAAGCGATCGA was mutated to *ACGCCTA*GAAGCGATCGA) under the same reaction conditions. The results showed no interaction between NtrC and the mutated *esrF* promoter (Fig. 6D), indicating that the motif is vital for the binding ability of NtrC to the *esrF* promoter.

### Negative regulation of *esrF* by NtrC is released in response to the high ammonium environment in rabbit colon.

**FIG 7:**
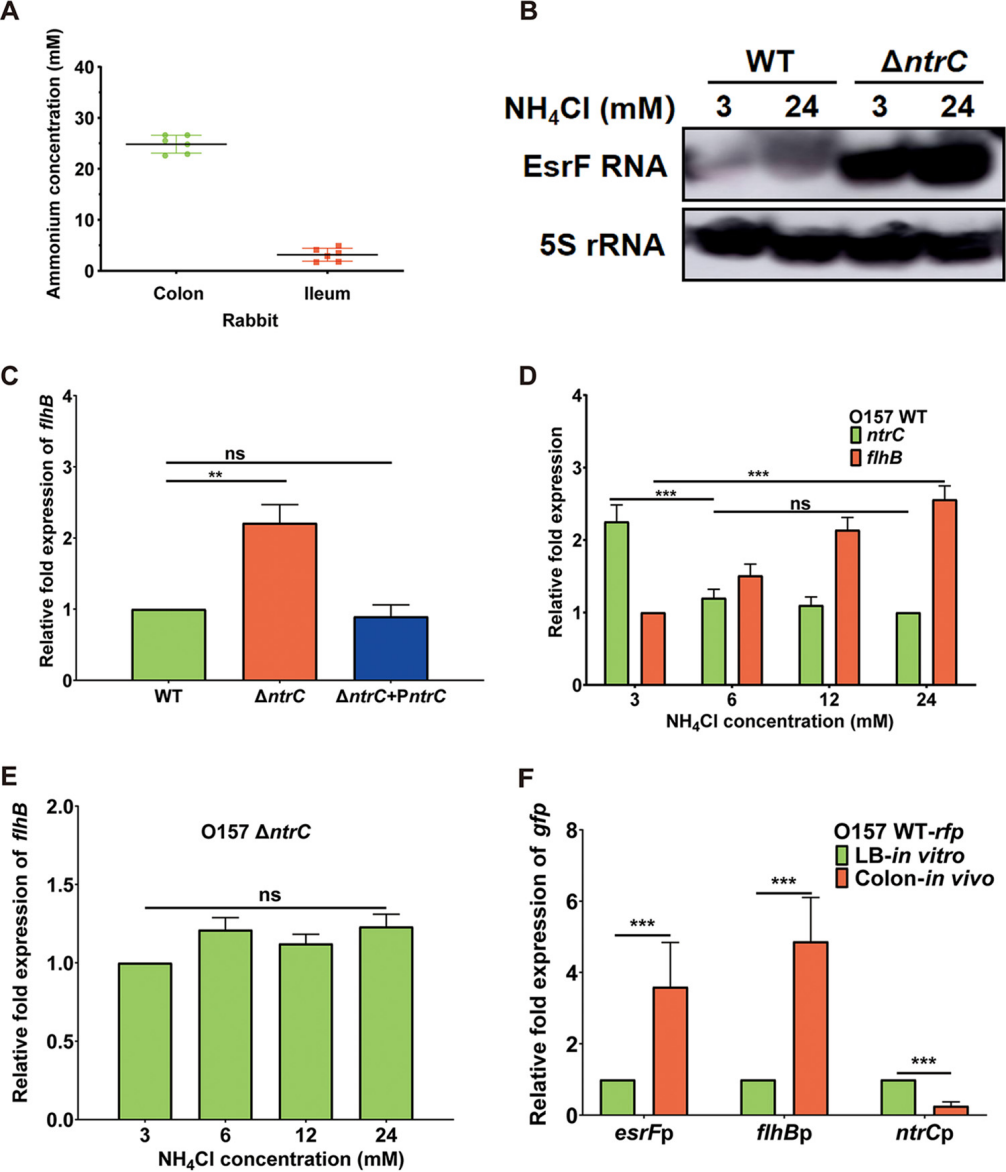
NtrC negatively regulates EsrF in different ammonium environments. (A) Ammonium concentrations of the rabbit colon and ileum were measured. (B) Northern blotting of EsrF in WT and Δ*ntrC* strains after treatment with 3 mM (low ammonium) and 24 mM (high ammonium) NH_4_Cl. (C) Expression of *flhB* in WT, Δ*ntrC*, and Δ*ntrC*+P*ntrC* strains analyzed using qRT-PCR. (D) *ntrC* and *flhB* expression in the WT strain with different concentrations of NH_4_Cl analyzed using qRT-PCR. (E) *flhB* in the Δ*ntrC* mutant expression with different concentrations of NH_4_Cl analyzed using qRT-PCR. (F) qRT-PCR detection of *gfp* expression to represent the *esrF*p, *flhB*p and *ntrC*p expressions of WT in the colon *in vivo* and LB medium-cultured samples. *rfp* was used as the internal control. The data are presented as means ± the SD (*n* = 3; ns, no significance; *, *P ≤ *0.05; **, *P ≤ *0.01; ***, *P ≤ *0.001). All *P* values were calculated using a Student *t* test.

Microenvironmental cues are sensed by various TCSs to regulate bacterial gene expression (30). For instance, the NtrC/B TCS can respond to a low ammonium ion concentration (3 mM) and regulate gene expression; however, it is inhibited under high ammonium ion concentrations (47). The average ammonium concentration in rabbit colon was found to be 24.1 mM (Fig. 7A), representing a high ammonium environment, which is in agreement with the previously reported concentration of 20.8 ± 8.0 mM in rabbit cecum (49).

We, therefore, investigated whether ammonium concentrations influence EsrF and *flhB* expression *in vitro*. Northern blotting results found that the abundance of O157 EsrF sRNA significantly increased in the presence of a high ammonium (24 mM) concentration compared to a low ammonium (3 mM) concentration (Fig. 7B; see also Fig. S4B). The qRT-PCR results further demonstrated that *flhB* expression increased significantly in Δ*ntrC* compared to the WT or Δ*ntrC*+P*ntrC* strains in DMEM (Fig. 7C). Further, the qRT-PCR results found that O157 *flhB* expression significantly increased with increasing ammonium concentrations (3, 6, 12, and 24 mM), while the reverse trend was observed for *ntrC* (Fig. 7D). However, the expression of *esrF* and *flhB* of the Δ*ntrC* strain exhibited no change between high-ammonium and low-ammonium conditions (Fig. 7B and E). This result indicates that repression of *esrF* by NtrC becomes released in a high-ammonium environment, resulting in upregulation of *flhB* expression *in vitro*.

In addition, *in vivo* qRT-PCR results showed that the *ntrC* expression of O157 WT strains in the colon of infected rabbits decreased significantly compared to that in the LB-cultured sample, indicating that *ntrC* expression was repressed in the high ammonium environment *in vivo* (Fig. 7F). Furthermore, in the colon of an infected rabbit, the expression of *esrF* and *flhB* of the O157 strain WT was enhanced compared to that in the LB medium-cultured sample. However, no difference was observed in the expression of *esrF* or *flhB* of the Δ*ntrC* strain in the colon of an infected rabbit and LB medium-cultured samples (see Fig. S4C). Together, these data indicate that O157 sensed the high ammonium concentrations in the rabbit colon and responded by turning off the negative regulation of *esrF* by NtrC, thus inducing the expression of *esrF* and *flhB* to promote its motility and virulence *in vivo*.

The expression of *glnA*, which is located upstream *esrF* in the genome, is also negatively regulated by NtrC (50). The qRT-PCR results showed that the expression of *glnA* in the Δ*ntrC* mutant increased significantly compared to that in WT and Δ*ntrC*+P*ntrC* strains (see Fig. S5A). Moreover, the expression of *glnA* in O157 significantly increased with increasing concentrations of ammonium (3, 6, 12, and 24 mM; see Fig. S5B). However, no changes were observed in the expression of *glnA* in the Δ*ntrC* mutant when exposed to different ammonium conditions (see Fig. S5C). The trend observed for *glnA* expression was the same as that for *esrF* under the regulation by NtrC in high-ammonium conditions, indicating that NtrC represses the expression of *esrF* and *glnA* through interacting with two unique independent promoters, respectively (Fig. 1D; see also Fig. S5).

10.1128/mBio.03605-20.5 FIG S5 *ntrC* negatively regulates *glnA* expression in different ammonium environments. (A) Expression of *glnA* in WT, Δ*ntrC*, and Δ*ntrC+PntrC* strains analyzed using qRT-PCR. (B) Expression of *glnA* in WT with different concentrations of NH_4_Cl analyzed using qRT-PCR. (C) Expression of *glnA* in the Δ*ntrC* mutant with different concentrations of NH_4_Cl analyzed using qRT-PCR. The data are presented as means ± the SD (ns, no significance; *, *P ≤ *0.05; **, *P ≤ *0.01; ***, *P ≤ *0.001). All *P* values were calculated using a Student *t* test. Download FIG S5, TIF file, 1.3 MB. Copyright © 2021 Jia et al. 2021 Jia et al. https://creativecommons.org/licenses/by/4.0/ This content is distributed under the terms of the Creative Commons Attribution 4.0 International license.

## DISCUSSION

O157 senses different host environmental signals, such as butyrate, fucose, and pH, via different TCSs that help to regulate virulence factor expression and promote bacteria colonization in the colon (51). For instance, a low biotin signal activates BirA-mediated expression of LEE genes to promote O157 virulence (52). In fact, most of the signal transduction pathways regulate O157 virulence by influencing the expression of LEE genes. However, the mechanisms by which microenvironmental cues in the colon regulate other non-LEE virulence genes remain unknown. In this study, we revealed a regulatory signal transduction pathway in which O157 utilizes a novel sRNA, EsrF, to sense high ammonium concentrations in the host colon to enhance bacterial motility and virulence. Specifically, the high ammonium concentration in the colon caused the repression of EsrF by NtrC to be lost, resulting in high levels of EsrF expression. EsrF then directly bound the 5′ UTR of *flhB* mRNA to promote *flhB* expression, which enhanced the expression of other flagellar genes to facilitate flagellar biosynthesis. This mechanism functions to significantly promote O157 motility and colonization in the colon.

Flagella play diverse roles in bacterial pathogenesis, such as in the migration to an optimal site in the host, colonization, and survival at the site of infection (53). They act as adhesins to promote host cell adhesion and microcolony formation (13). Flagella were identified as adhesins when a *fliC* mutation was found to reduce EHEC virulence (54). For pathogens, it is necessary to upregulate the expression of flagellar genes during the early stages of infection. Here, we discovered that EsrF promoted O157 motility and host cell adhesion during these early stages. FlhDC and FliA are well-known master flagellar transcriptional activators in E. coli that regulate other flagellar genes (16). In our study, we found that EsrF positively regulated flagellar gene expression via FlhB in O157, which upregulated the expression of *flhD*, *flhC*, *fliA*, and *fliC.* This observation is consistent with the findings of a previous report that FlhB can regulate flagellar gene expression in Listeria monocytogenes (17). However, the detailed mechanism of how FlhB regulates flagellar genes is unknown.

The potential mechanism underlying the increased *flhB* mRNA abundance induced by EsrF-*flhB* mRNA interaction is likely related to the loss of Rho termination in O157. In bacterial cells, the Rho factor mediates termination of transcription at specific sites, which can be relieved by the Rho-specific inhibitor bicyclomycin (BCM) (55). Certain sRNAs in bacteria can anneal within 5′ UTRs to inhibit premature Rho termination and activate the expression of corresponding genes (27). Hence, we analyzed the sequencing reads in the *flhB* 5′ UTR region from the published raw data generated by Sedlyarova et al. from an RNA-seq analysis on E. coli, with or without BCM treatment (27) (see Fig. S6A). The upregulated expression and released termination within *flhB* 5′ UTRs in response to BCM treatment was found to occur between sites 0 and 100 (counted from *flhB* translational start site), indicating that Rho factor targets are located within *flhB* 5′ UTRs. Moreover, our identified EsrF binding sites were determined to be located at *flhB* 5′ UTR positions −82 to −97, within the Rho termination region. Besides, we analyzed the transcription of *flhB* open reading frame (ORF) in O157 WT, with or without BCM treatment, by qRT-PCR assays and found that *flhB* expression in O157 WT with BCM treatment exhibited a 2.2-fold increase compared to that in O157 WT without BCM treatment (see Fig. S6B). Taken together, these results indicate that the interaction between EsrF with the *flhB* 5′ UTR may release the Rho-dependent termination within the *flhB* 5′ UTR, leading to increased *flhB* expression.

10.1128/mBio.03605-20.6 FIG S6 EsrF-*flhB* mRNA interaction increases *flhB* mRNA abundance related to relieved Rho-termination. (A) Transcriptomic coverage plots for *flhB* 5′ UTR before (up) and after (down) treatment with bicyclomycin (BCM). (B) Transcription of *flhB* ORF in O157 WT without BCM (WT-BCM) or with BCM (WT+BCM) treatment (15 min, 50 μg/ml) analyzed using qRT-PCR. The data are presented as means ± the SD (ns, no significance; *, *P ≤ *0.05; **, *P ≤ *0.01; ***, *P ≤ *0.001). All *P* values were calculated using a Student *t* test. Download FIG S6, TIF file, 1.7 MB. Copyright © 2021 Jia et al. 2021 Jia et al. https://creativecommons.org/licenses/by/4.0/ This content is distributed under the terms of the Creative Commons Attribution 4.0 International license.

Inorganic N, produced by colonic microflora and digested food, is the secondmost important element after carbon in organisms and is a crucial constituent of proteins, nucleic acids, and cell walls. Various types of inorganic and organic N are assimilated by mammals, including ammonium (NH_4_^+^), nitrate (NO_3_^–^), nitrite (NO_2_^–^), urea, purine, and amino acids. Ammonium, widely used in various food additives, is the most effective N source for bacteria, which is assimilated directly into important biosynthetic reactions (47). Bacterial N metabolism is mainly regulated by the NtrC/B TCS, which regulates approximately 100 genes, mainly for N assimilation under N starvation (56). During N limitation, the NtrB/C TCS positively modulates Pseudomonas aeruginosa virulence by producing rhamnolipids (57) and exopolyphosphatase (Ppx) (58, 59). NtrC-regulated exopolysaccharides are involved in biofilm formation and the pathogenic interaction of Vibrio vulnificus (60). However, NtrB/C has not been reported to be related to the virulence of pathogenic E. coli. In this study, we have demonstrated that NtrC bound directly to the *esrF* promoter, sensing high ammonium concentrations in the colon to induce O157 motility and host cell adhesion.

Genome sequence analysis for 226 representative E. coli strains revealed that *esrF* is highly conserved and ubiquitous in different E. coli strain pathotypes. Specifically, the strains containing *esrF* include EHEC strains O157:H7, O157 Sakai, O157 Xuzhou21, enteropathogenic E. coli (EPEC) strains O55:H7, neonatal meningitis-associated E. coli strain RS218, urinary pathogenic E. coli strain CFT073, avian pathogenic E. coli strain LF82, as well as other clinical isolates of E. coli strains (see Fig. S7A and Table S2 in the supplemental material). However, *esrF* is absent in nonpathogenic E. coli strains, such as K-12, indicating that EsrF is a horizontal transfer-acquired regulatory sRNA of pathogenic E. coli, while *esrF* may be associated with the evolution from nonpathogenic E. coli to pathogenic strains.

10.1128/mBio.03605-20.7 FIG S7 EsrF is widely found in pathogenic Escherichia coli. (A) Maximum-likelihood tree constructed in PhyML based on 1,112 single-copy core genes shared by 226 E. coli strains. The strains in different clades containing *esrF* are shown in different colors. (B) *flhB* expression compared between three representative pathogenic E. coli strains, G1352 (EHEC O26:H11), G4943 (EPEC O55:H7), and G1474 (EPEC O86:H34) with Δ*esrF* ortholog mutants. The data are presented as means ± the SD (ns, no significance; *, *P ≤ *0.05; **, *P ≤ *0.01; ***, *P ≤ *0.001). All *P* values were calculated using a Student *t* test. Download FIG S7, TIF file, 2.1 MB. Copyright © 2021 Jia et al. 2021 Jia et al. https://creativecommons.org/licenses/by/4.0/ This content is distributed under the terms of the Creative Commons Attribution 4.0 International license.

10.1128/mBio.03605-20.9 TABLE S2 Prevalence of *esrF* among different E. coli strains. Download Table S2, DOCX file, 0.02 MB. Copyright © 2021 Jia et al. 2021 Jia et al. https://creativecommons.org/licenses/by/4.0/ This content is distributed under the terms of the Creative Commons Attribution 4.0 International license.

To investigate whether homologous EsrF in other flagellated E. coli strains also regulate *flhB* expression, we selected three representative strains (EHEC O26:H11, EPEC O55:H7, and EPEC O86:H34) and deleted the *esrF* orthologous genes to construct corresponding deletion strains. Compared to the corresponding WT strains, the *flhB* expression level of these mutants was significantly reduced (see Fig. S7B), indicating that the EsrF-mediated mechanism for *flhB* regulation may be present in various pathogenic E. coli strains.

## MATERIALS AND METHODS

### Bacterial strains, plasmids, and primers.

The bacterial strains, plasmids, and primers used in this study are listed in Table S3 in the supplemental material. Mutant strains were generated by substitution of the chloramphenicol or kanamycin resistance genes in plasmid pKD3 or pKD4 with sRNA or the relevant genes, respectively, by using the λ Red recombinase system (61). The complementary strains were constructed by cloning the ORF and upstream promoter sequence of corresponding genes into the vector, pBluescript II SK(+) (62). The pET-*ntrC* strain was generated by cloning the *ntrC* gene into the downstream region of the His tag element in plasmid pET-28a. All the resulting clones were verified by PCR amplification and DNA sequencing.

10.1128/mBio.03605-20.10 TABLE S3 Bacterial strains, plasmids, and primers used in this study. Download Table S3, DOCX file, 0.03 MB. Copyright © 2021 Jia et al. 2021 Jia et al. https://creativecommons.org/licenses/by/4.0/ This content is distributed under the terms of the Creative Commons Attribution 4.0 International license.

### Bacterial growth and cell culture conditions.

O157 and its derivatives were cultured in LB medium or DMEM to simulate *in vivo* colon conditions. IPTG (isopropyl-β-d-thiogalactopyranoside) and antibiotics were used whenever necessary. After adding overnight subcultures into 96-well plates, the growth curves of all strains were measured over 24 h by using a plate reader. Each experiment was independently performed three times. The HeLa (ATCC CCL-2) cell line was grown in DMEM supplemented with 10% fetal bovine serum (Gibco) and penicillin-streptomycin and incubated at 37°C in a 5% CO_2_-containing atmosphere.

### RNA isolation and qRT-PCR.

To test the influence of virulence genes, strains were grown in DMEM up to the exponential phase (optical density at 600 nm [OD_600_] ∼0.6). To test the influence of ammonium concentrations on gene expression, strains were grown in M9 medium under low-ammonium (3 mM) and high-ammonium (24 mM) conditions. To quantify gene expression *in vivo*, the constitutively expressed red fluorescence protein (*rfp*) gene, inserted into the O157 chromosome, was used as the reference control for normalization. We also constructed a plasmid containing the *esrF*, *flhB*, and *ntrC* promoter transcriptionally fused to a green fluorescent protein (*gfp*) gene and introduced it into O157 cells with *rfp*. The expression of *esrF*, *flhB*, and *ntrC* was then indirectly determined by qRT-PCR analysis for the expression of *gfp* (to avoid interference of *esrF*, *flhB*, and *ntrC* homologs from intestinal commensal bacteria). The overnight cultures were separated into one sample for RNA extraction and another for oral gavage to a rabbit, the colon of which was dissected for RNA extraction. Total RNA was extracted using the TRIzol LS reagent (catalog no. 10296028; Invitrogen, Carlsbad, CA) and treated with RNase-free DNase I to eliminate genomic DNA contamination. qRT-PCR was performed as follows. Briefly, 2 μg of diluted, extracted RNA was converted to cDNA with Superscript IV VILO Master Mix (Invitrogen). Validated primers (see Table S3) and SYBR green were added to the cDNA, and the mixture was amplified using QuantStudio 5 (Applied Biosystems). Data were collected using QuantStudio Real-Time PCR Software v1.3, normalized to endogenous *rpoA* levels, and analyzed using the comparative critical threshold (*C_T_*) method. Each experiment was performed in triplicate.

### RNA-seq and other bioinformatic tools.

Total RNA was purified by using an RNeasy minikit (Qiagen) and a Ribo-Zero rRNA removal kit (Epicentre Biotechnologies, RZNB1056). Libraries of RNA samples were generated by using an NEBNext R Ultra Directional RNA Library Prep kit for Illumina R (NEB). The libraries were sequenced using an Illumina HiSeq platform to generate paired-end reads. To search for potential sRNAs of O157, plot sequence reads were aligned to the reference sequence of O157 usin*g* Burrows-Wheeler Aligner software and SAMtools. TargetRNA2 was used to predict target genes of sRNA by searching the 5′ regions (ca. nt −120 to +20 relative to the start codon) of all O157 gene mRNAs for potential RNA duplex formation sequences (43).

### Northern blotting.

Northern blotting was performed using the DIG Northern Starter kit (Roche). RNA was separated on a 1.2% agarose gel containing 37% formaldehyde. The size of the RNA was determined by comparing it to the RNA Century-Plus Markers (Invitrogen). Gels were electroblotted onto Brightstar Plus nylon membranes (Applied Biosystems) and immobilized at 120°C for 30 min. Cross-linked membranes were prehybridized for 30 min in digoxigenin (DIG) Easy Hyb buffer. Single-stranded RNA probes were labeled with DIG added to fresh Easy Hyb buffer, and the blots were incubated with hybridization buffer overnight. After high- and low-stringency washes, the blots were further washed using the Wash and Block Buffer, and CDP-Star (Roche) was added as the substrate. Hybridization signals were visualized by using Amersham Imager 680, 5S rRNA being used as an internal control. ImageJ software was used to measure the band intensities. The specific DIG-labeled RNA probes used in the Northern blotting are presented in Table S3.

### Exonuclease digestion of RNA.

RNA samples were prepared from O157 WT grown overnight in LB. Treatment of RNA samples with terminator 5-monophosphate-dependent exonuclease (TEX; Epicentre Biotechnologies) was performed as described previously (38). Total RNA (10 μg/sample) samples were incubated with TEX in a final 20-μl reaction volume containing 2 μl of 10× reaction buffer (500 mM Tris-HCl [pH 8.0], 20 mM MgCl_2_, 1 M NaCl), 1 μl of RNasin (40 U; Promega), and 1 μl of TEX for 1 h at 30°C. The reaction mixture was then purified and analyzed by Northern blotting as described above.

### 5′ and 3′ RACE assay.

RACE was performed with the 5′/3′-RACE system (Invitrogen). For 5′-RACE, 5 μg of RNA was reverse transcribed using an sRNA-specific antisense primer and SuperScript reverse transcriptase (Invitrogen). cDNA was then purified, dC tailed, and used as a template in a PCR with the Abridged Anchor Primer (AAP) and a nested gene-specific primer. 3′-RACE was performed by ligating a poly(A) tail using a poly(A) polymerase tailing kit (Epicentre Biotechnologies) before reverse transcription. Specific cDNAs were then directly amplified by PCR using an anchor primer (AP) that targets the poly(A) tail region and a gene-specific primer that anneals to a region of known sRNA sequence. PCR products were cloned into the pEASY-T1 simple cloning vector (TransGen) before sequencing.

### Cell adhesion assay.

Subcultured bacteria were grown in DMEM until the OD_600_ was 0.6; the OD_600_ was then adjusted to 0.3 with DMEM. Next, bacterial cells (2 ml each well) were added to HeLa cell monolayers in 6-well plates at a multiplicity of infection (MOI) of 100. To avoid motility-related effects, all bacteria were centrifuged onto the surface of cells and incubated for 1 h at the early infection stage. The cells were then washed, lysed, and plated on LB agar plates. The adhesion rates were calculated as percentages of the number of bacteria recovered relative to the total bacteria inoculated. All assays were performed in triplicate.

### Fluorescein actin staining.

Fluorescence actin staining was performed as previously described (63). Human cervical adenocarcinoma (HeLa) cells were grown on coverslips to 60% confluence and infected by bacteria as described for the cell adhesion assay. After infection, the coverslips were washed and fixed with 4% formaldehyde, permeabilized with 0.2% Triton X-100, and treated with fluorescein isothiocyanate (FITC)-labeled phalloidin to visualize actin accumulation, followed by propidium iodide (PI) to visualize the DNA. The coverslips were mounted on slides and observed under a Leica TCS SP8 microscope. The AE lesions formed in each cell were enumerated for 50 HeLa cells; three slides were observed for each strain in each experiment.

### Infant rabbit model of O157 infection.

To prepare the inoculum, bacteria were grown overnight in LB broth, harvested by centrifugation, resuspended in sterile phosphate-buffered saline, and adjusted to a cell density of ∼10^9^ CFU ml^−1^. Three-day-old New Zealand White rabbits were intragastrically inoculated with ∼5 × 10^8^ CFU/90 g (body weight) of WT or mutant in a 0.5-ml inoculum, which was followed by the administration of 2.5 ml of sterile 0.85% saline solution to ensure delivery of the entire inoculum. Rabbits were euthanized 48 h postinfection. At necropsy, the intestinal tract from the duodenum to the anus was removed to collect samples for microbiologic analyses. Rabbit competition experiments were performed as follows: Δ*flhB* and Δ*esrF* Δ*flhB* mutant strains, both in logarithmic phase, were mixed at a 1:1 ratio for oral inoculation of rabbits, as described above. The CI value of the Δ*flhB* mutant versus the Δ*esrF* Δ*flhB* mutant was calculated. All experiments were performed in triplicate.

### Motility assay and transmission electron microscopy.

Overnight cultures were adjusted to an OD_600_ of 0.2, and then 5 μl was stab inoculated onto 0.35% semi-LB-agar plates. The agar plates were incubated at 25°C for 12 h, and the diameter of the swimming zone around the inoculation site was measured. TEM was performed to observe the flagellum morphology on the surface of O157, as previously described (17, 64). The strains were cultured in LB plates overnight. Single colonies were resuspended in 50 μl of medium; 10 μl was then dropped onto and absorbed for 5 min into carbon-stabilized Formvar supports on 200-mesh copper grids. Cells were then stained by submerging the grids for 5 min in 2% (wt/vol) uranyl acetate and imaged using a JEM-1400 Plus transmission electron microscope (JEOL) operating at 100 kV and fitted with a high-sensitivity real-time charge-coupled device camera. All strains were tested in triplicate, and the number of flagella were counted per 30 bacteria.

### Purification of NtrC protein and protein-DNA EMSA.

NtrC-6×His protein was expressed using BL21(DE3)-containing pET-NtrC and purified by using a MagneHis protein purification system (Promega). Protein-DNA EMSAs were performed as described previously (65). EsrF promoter regions were amplified and purified. NtrC protein shift assays were performed by incubating EsrF promoter fragments (15 nM) at 37°C for 30 min with various concentrations of NtrC-6×His protein (0 to 3 μM) in a 20-μl solution containing bandshift buffer (24 mM Tris-HCl [pH 7.5], 80 mM NaCl, 0.1 mM EDTA, 1 mM dithiothreitol [DTT]). In addition, 30 mM acetyl phosphate (AcP) was added as the donor to phosphorylate NtrC to NtrC-P. The samples were loaded on an 8% polyacrylamide gel. The DNA fragments were stained for 10 min with StarGreen (Genstar) and visualized by UV transillumination. ImageJ software was used to measure the band intensities.

### Dye primer-based DNase I footprinting assay.

The EsrF promoter (7.5 nM) was amplified with a forward primer (with a 6-FAM modification at the 5′ end) and a reverse primer and then incubated with 1 μM NtrC and 1 μM bovine serum albumin (BSA) in bandshift buffer. The protein-DNA mixtures were then partially digested with 0.05 U of DNase I for 5 min at 25°C, quenched by using 0.25 mol/liter EDTA, and purified. Control samples were prepared with 2 μM BSA. All genotype samples were analyzed using the ABI 3730 DNA analyzer.

### *In vitro* transcription and RNA-RNA EMSA.

The *in vitro* transcription DNA templates of EsrF (+), EsrF (−; positive control), and *flhB* mRNA were PCR amplified using O157 genome as the template. Then, DNA templates were transcribed into EsrF (+), EsrF (−), and *flhB* mRNA using the T7 high-efficiency transcription kit (TransGen) and purified by using the EasyPure RNA purification kit (TransGen). The purified RNA was checked in an 8% Tris-Acr-urea gel. An RNA-RNA EMSA (REMSA) was performed to verify the interaction of RNAs, as previously described (23, 66). REMSAs were performed with EsrF (+, 0.5, 1, 2, and 4 μM), EsrF (−, positive control, 1 μM) and *flhB* mRNA (1 μM), 10× REMSA binding buffer (100 mM HEPES [pH 7.3], 200 mM KCl, 24 mM MgCl_2_, and 24 mM DTT), and RNase-free water (Beyotime). The reaction mixtures were incubated for 2 min at 85°C and then at 37°C for 30 min. RNA was separated by 8% Native PAGE using a Native-PAGE preparation kit (Sangon), stained with SYBR Gold nucleic acid gel stain (Invitrogen) for 10 min, and visualized under UV light. ImageJ software was used to measure band intensities.

### Ammonium concentration measurements.

The colons from standard 3-day-old New Zealand White rabbits were collected, washed, and homogenized using a Tissueprep Lyser. The samples were pelleted, and the supernatants were collected. The ammonium concentrations of the serially diluted supernatants were measured by using a Quantofix ammonium test kit (Merck Millipore, UK) according to the manufacturer’s instructions.

### Phylogenetic analysis.

Orthologous groups were identified using OrthoFinder (67) by which all nucleotide sequences were compared using a BLASTN all-against-all search with an E value cutoff of <10^−4^. Nucleotide sequences used to construct the phylogenetic tree were aligned in MAFFT (68), and a maximum-likelihood tree was constructed in PhyML and FigTreev1.4.3 based on the GTR model of nucleotide substitution with c-distributed rates among sites.

### Ethics statement.

All animal experiments were carried out according to the standards set forth in the *Guide for the Care and Use of Laboratory Animals* published by the Institute of Laboratory Animal Resources of the National Research Council (United States). The experimental protocols were approved by the Institutional Animal Care Committee at Nankai University. Every effort made was to minimize animal suffering and to reduce the number of animals used.

### Statistical analysis.

Statistical analysis was performed using MedCalc 15.6; figures were drawn by GraphPad Prism 8 and integrated by using Adobe Illustrator CC 2020. All data are expressed as means ± the standard deviations (SD). Differences between two groups were evaluated using a two-tailed Student *t* test or a Mann-Whitney U test. *P* values of *≤*0.05, 0.01, or 0.001 were considered statistically significant (∗), highly significant (∗∗), or extremely significant (∗∗∗), respectively.
