# Supplementary material for: A Novel Small RNA Promotes Motility and Virulence of Enterohemorrhagic Escherichia coli O157:H7 in Response to Ammonium
Source: mBio. 2021 Mar 9;12(2):e03605-20. doi: 10.1128/mBio.03605-20 (PMC8092317; doi:10.1128/mBio.03605-20)
Supplement: TABLE S1 [file mBio.03605-20-st001.docx]

**Table S1A.** TargetRNA2 used to predict 53 putative target genes of EsrF.

| **TargetRNA2** | | | | |
| --- | --- | --- | --- | --- |
| **Rank** | **Gene** | **Synonym** | **Energy** | **P_value_^a^** |
| 1 | - | *Z6067* | -17.06 | 0.000 |
| 2 | *prfH* | *Z0297* | -13.67 | 0.002 |
| 3 | - | *Z0040* | -12.91 | 0.004 |
| 4 | - | *Z1330* | -12.21 | 0.006 |
| 5 | - | *Z3672* | -11.73 | 0.009 |
| 6 | *aceE* | *Z0124* | -11.72 | 0.009 |
| 7 | *speE* | *Z0131* | -11.38 | 0.011 |
| 8 | *dkgB* | *Z0229* | -11.17 | 0.012 |
| 9 | - | *Z1331* | -11.03 | 0.013 |
| 10 | - | *Z3241* | -10.87 | 0.015 |
| 11 | - | *Z2967* | -10.7 | 0.016 |
| 12 | - | *Z1210* | -10. 62 | 0.017 |
| 13 | - | *Z1650* | -10. 62 | 0.017 |
| 14 | *malY* | *Z2627* | -10.59 | 0.017 |
| 15 | *purT* | *Z2901* | -10.56 | 0.017 |
| 16 | *ilvl* | *Z0087* | -10.37 | 0.019 |
| 17 | - | *Z5691* | -10.27 | 0.020 |
| 18 | *dcuC* | *Z0766* | -10.22 | 0.021 |
| 19 | *nagA* | *Z0824* | -10.19 | 0.021 |
| 20 | - | *Z1780* | -10.14 | 0.022 |
| 21 | *ybjX* | *Z1112* | -10.14 | 0.022 |
| **22** | ***flhB*** | ***Z2934*** | **-9.76** | **0.025** |
| 23 | *exoP* | *Z6080* | -9.65 | 0.028 |
| 24 | - | *Z2790* | -9.46 | 0.030 |
| 25 | *eutG* | *Z3709* | -9.42 | 0.031 |
| 26 | *ybfF* | *Z0835* | -9.3 | 0.033 |
| 27 | *-* | *Z0244* | -9.19 | 0.034 |
| 28 | *treR* | *Z5851* | -9.07 | 0.036 |
| 29 | *ycgC* | *Z1969* | -8.97 | 0.038 |
| 30 | *yjjY* | *Z6005* | -8.96 | 0.038 |
| 31 | *-* | *Z2366* | -8.85 | 0.040 |
| 32 | *sanA* | *Z3399* | -8.84 | 0.040 |
| 33 | *-* | *Z2694* | -8.83 | 0.040 |
| 34 | *exoO* | *Z2037* | -8.77 | 0.041 |
| 35 | *-* | *Z4217* | -8.76 | 0.041 |
| 36 | *yehB* | *Z3277* | -8.76 | 0.041 |
| 37 | *-* | *Z1881* | -8.63 | 0.043 |
| 38 | *cbpA* | *Z1418* | -8.62 | 0.043 |
| 39 | *cutF* | *Z0204* | -8.44 | 0.043 |
| 40 | *-* | *Z2043* | -8.44 | 0.044 |
| 41 | *-* | *Z6032* | -8.43 | 0.044 |
| 42 | *ybgF* | *Z0910* | -8.42 | 0.044 |
| 43 | *yfhL* | *Z3842* | -8.42 | 0.045 |
| 44 | *deoD* | *Z5986* | -8.41 | 0.045 |
| 45 | *-* | *Z2045* | -8.41 | 0.046 |
| 46 | *-* | *Z0990* | -8.40 | 0.046 |
| 47 | *rseA* | *Z3854* | -8.40 | 0.047 |
| 48 | *ompX* | *Z1036* | -8.39 | 0.047 |
| 49 | *-* | *Z2365* | -8.39 | 0.048 |
| 50 | *-* | *Z2124* | -8.37 | 0.048 |
| 51 | *fliY* | *Z3010* | -8.36 | 0.049 |
| 52 | *-* | *Z6076* | -8.35 | 0.049 |
| 53 | *-* | *Z2042* | -8.34 | 0.049 |

a: P_value_<0.05.

**Table S1B** Differently expressed genes of the O157 Δ*esrF* transcriptome.

| **O157 Δ*esrF* transcriptome** | | | | | |
| --- | --- | --- | --- | --- | --- |
| **Gene name** | **Function** | **Fold change^a^** | | | **P_value_^b^** |
| **187 downregulated genes:** | | | | |  |
| *Z6081* | Putative excisionase of cryptic prophage CP-933P | | | >-10.1 | 0.002863891 |
| *Z6053* | Putative holin protein encoded by cryptic prophage CP-933P | | | >-9.7 | 5.00E-12 |
| *Z6048* | Unknown protein encoded by cryptic prophage CP-933P | | >-9.6 | | 0.000290613 |
| *Z6026* | Unknown protein encoded by cryptic prophage CP-933P | | >-9.6 | | 2.82E-05 |
| *Z6005* | Hypothetical protein | | >-9.6 | | 2.88E-11 |
| *Z5852* | Hypothetical protein | | >-9.5 | | 2.22E-13 |
| *Z5754* | Putative toxin of osmotically regulated toxin-antitoxin system associated with programmed cell death | | >-9.5 | | 2.82E-05 |
| *Z5489* | Hypothetical protein | | >-9.5 | | 2.78E-10 |
| *Z5432* | Hypothetical protein | | >-9.5 | | 2.56E-11 |
| *Z5166* | *ilvBN* operon leader peptide | | >-9.4 | | 6.15E-16 |
| *Z4723* | Hypothetical protein | | >-9.4 | | 1.13E-26 |
| *Z4708* | Hypothetical protein | | >-9.4 | | 9.96E-17 |
| *Z4601* | Hypothetical protein | | >-9.4 | | 0.009223959 |
| *Z4398* | Hypothetical protein | | >-9.4 | | 7.45E-07 |
| *Z4367* | Hypothetical protein | | >-9.3 | | 0.056521739 |
| *Z4192* | Hypothetical protein | | >-9.3 | | 9.64E-05 |
| *Z4071* | Hypothetical protein | | >-9.3 | | 0.000390825 |
| *Z4062* | Hypothetical protein | | >-9.3 | | 1.45E-05 |
| *Z4045* | Hypothetical protein | | >-9.3 | | 2.29E-08 |
| *Z3924* | Partial Putative transposase | | >-9.2 | | 1.17E-05 |
| *Z3464* | Hypothetical protein | | >-9.2 | | 9.96E-17 |
| *Z3335* | Unknown protein encoded within prophage CP-933V | | >-9.2 | | 2.94E-06 |
| ***Z2934*** | **Flagellar membrane protein FlhB** | | **>-9.2** | | **0.021900161** |
| *Z3165* | Hypothetical protein | | >-9.2 | | 1.66E-12 |
| *Z3127* | Unknown protein encoded within prophage CP-933U | | >-9.1 | | 0.0285714286 |
| *Z3118* | Unknown protein encoded within prophage CP-933U | | >-9.1 | | 0.001006409 |
| *Z3048* | Hypothetical protein | | >-9.1 | | 6.37E-13 |
| *Z2970* | Putative regulator for prophage CP-933T | | >-9.1 | | 4.00E-13 |
| *Z2838* | Hypothetical protein | | >-9.1 | | 2.78E-28 |
| *Z2618* | Hypothetical protein | | >-9.0 | | 8.64E-08 |
| *Z2553* | Hypothetical protein | | >-9.0 | | 5.19E-06 |
| *Z2480* | Phage shock protein B | | >-9.0 | | 4.45E-27 |
| *Z2478* | Phage shock protein | | >-9.0 | | 5.25E-12 |
| *Z2406* | Unknown protein encoded within prophage CP-933R | | >-9.0 | | 3.52E-19 |
| *Z2374* | Putative holin protein of prophage CP-933R | | >-9.0 | | 4.66E-07 |
| *Z2343* | Partial Putative outer membrane protein Lom precursor encoded by prophage CP-933R | | >-9.0 | | 9.64E-05 |
| *Z2255* | Unknown protein associated with Rhs element | | >-8.9 | | 7.45E-07 |
| *Z2254* | Partial H repeat-associated protein of Rhs element | | >-8.9 | | 0.042578711 |
| *Z2181* | Hypothetical protein | | >-8.9 | | 0.0090133159 |
| *Z2156* | Hypothetical protein | | >-8.9 | | 0.0171885536 |
| *Z2122* | Putative holin protein of prophage CP-933O | | >-8.9 | | 0.0052173913 |
| *Z2055* | Unknown protein encoded by prophage CP-933O | | >-8.9 | | 0.0283652797 |
| *Z1924* | Unknown protein encoded by prophage CP-933X | | >-8.8 | | 0.0211790394 |
| *Z1912* | Unknown protein encoded by prophage CP-933X | | >-8.8 | | 0.0052173913 |
| *Z1905* | Unknown protein encoded by prophage CP-933X | | >-8.8 | | 0.00658385093 |
| *Z1891* | Putative tail component of prophage CP-933X | | >-8.8 | | 0.014686812 |
| *Z1879* | Putative envelope protein of prophage CP-933X | | >-8.8 | | 0.048635926 |
| *Z1841* | Unknown protein encoded by prophage CP-933C | | >-8.7 | | 0.041361276 |
| *Z1839* | Unknown protein encoded by prophage CP-933C | | >-8.7 | | 0.042173913 |
| *Z1782* | Unknown protein encoded by prophage CP-933N | | >-8.7 | | 0.0485507246 |
| *Z1624* | hypothetical protein | | >-8.7 | | 0.0305221314 |
| *Z1620* | hypothetical protein | | >-8.6 | | 0.0108432879 |
| *Z1576* | hypothetical protein | | >-8.6 | | 0.0129436724 |
| *Z1504* | Amino terminal fragment of WrbA | | >-8.6 | | 0.045015131 |
| *Z1468* | putative lysis protein S of bacteriophage BP-933W | | >-8.6 | | 0.0146338567 |
| *Z1420* | hypothetical protein | | >-8.5 | | 0.0228157586 |
| *Z1405* | cold shock-like protein | | >-8.5 | | 0.0148269996 |
| *Z1209* | hypothetical protein | | >-8.5 | | 0.0442578711 |
| *Z1151* | hypothetical protein | | >-8.5 | | 0.042173913 |
| *Z0968* | unknown protein encoded by prophage CP-933K | | >-8.4 | | 0.0238790282 |
| *Z0653* | hypothetical protein | | >-8.4 | | 0.0219276674 |
| *Z0583* | hypothetical protein | | >-8.4 | | 0.0148529576 |
| *Z0416* | putative ATP-binding component of transport system | | >-8.4 | | 0.0126130427 |
| *Z0387* | hypothetical protein | | >-8.3 | | 0.0103177555 |
| *Z0344* | hypothetical protein | | >-8.3 | | 0.042851146 |
| *Z0110* | hypothetical protein | | >-8.3 | | 0.0442578711 |
| *Z2187* | hypothetical protein | | >-8.2 | | 0.0126130427 |
| *Z5189* | hypothetical protein | | >-8.2 | | 0.0148529576 |
| *Z4894* | universal stress protein UspB | | >-8.2 | | 0.0109694602 |
| *Z4937* | hypothetical protein | | >-8.1 | | 0.0238790282 |
| *Z2661* | superoxide dismutase precursor sod (Cu-Zn) | | >-8.1 | | 0.037294686 |
| *Z4452* | hypothetical protein | | >-8.0 | | 0.0426708075 |
| *Z2884* | hypothetical protein | | >-8.0 | | 0.0305221314 |
| *Z2421* | hypothetical protein | | >-8.0 | | 0.049678526 |
| *Z4802* | putative ATP-dependent DNA helicase (together with adjacent 3 orfs) | | >-8.0 | | 0.238790282 |
| *Z2761* | catalase; hydroperoxidase HPII(III) | | >-7.9 | | 0.039151782 |
| *Z3768* | hypothetical protein | | >-7.9 | | 0.0458385093 |
| *Z1437* | putative Bet recombination protein of bacteriophage BP-933W | | >-7.9 | | 0.042173913 |
| *Z3815* | hypothetical protein | | >-7.8 | | 0.0430641822 |
| *Z5148* | hypothetical protein | | >-7.8 | | 0.0187025842 |
| *Z4453* | hypothetical protein | | >-7.8 | | 0.0426708075 |
| *Z4356* | oxidoreductase | | >-7.7 | | 0.018037156 |
| *Z1485* | unknown protein encoded by bacteriophage BP-933W | | >-7.7 | | 0.042173913 |
| *Z2972* | unknown protein encoded by prophage CP-933T | | >-7.5 | | 0.0111592322 |
| *Z6038* | putative structural component of cryptic prophage CP-933P | | >-7.5 | | 0.0333680779 |
| *Z3965* | hypothetical protein | | >-7.4 | | 0.0238790282 |
| *Z0606* | glutaminase | | >-7.4 | | 0.0148529576 |
| *Z6066* | putative exclusion protein ren of cryptic prophage CP-933P | | >-7.3 | | 0.049474366 |
| *Z3964* | hypothetical protein | | >-7.3 | | 0.039271064 |
| *Z3940* | hypothetical protein | | >-7.2 | | 0.048174706 |
| *Z3370* | unknown protein encoded within prophage CP-933V | | >-7.2 | | 0.014806642 |
| *Z2823* | hypothetical protein | | >-7.2 | | 0.026703115 |
| *Z4929* | putative ARAC-type regulatory protein | | >-7.1 | | 0.044095619 |
| *Z3386* | oxidoreductase | | >-7.1 | | 0.037294686 |
| *Z5695* | phosphonate metabolism | | >-7.0 | | 0.0146764611 |
| *Z1007* | hypothetical protein | | >-7.0 | | 0.03515516 |
| *Z0859* | deoxyribodipyrimidine photolyase (photoreactivation) | | >-6.9 | | 0.014806642 |
| *Z2230* | 30S ribosomal subunit protein S22 | | >-6.9 | | 0.0122481004 |
| *Z2554* | putative structural proteins | | >-6.9 | | 0.0238790282 |
| *Z3380* | putative transport system permease protein | | >-6.8 | | 0.026618956 |
| *Z1686* | acidic protein suppresses mutants lacking function of protein export | | >-6.8 | | 0.037294686 |
| *Z4451* | hypothetical protein | | >-6.8 | | 0.0266666667 |
| *Z3379* | putative transport system permease protein | | >-6.7 | | 0.0266666667 |
| *Z5490* | hypothetical protein | | >-6.7 | | 0.0146764611 |
| *Z2359* | partial putative capsid structural protein of prophage CP-933R | | >-6.6 | | 0.042908497 |
| *Z6040* | putative head-tail adaptor of cryptic prophage CP-933P | | >-6.6 | | 0.040452122 |
| *Z5337* | hypothetical protein | | >-6.6 | | 0.023957324 |
| *Z3366* | putative recombination protein Bet of prophage CP-933V | | >-6.5 | | 0.0423809524 |
| *Z3260* | fructose-bisphosphate aldolase | | >-6.5 | | 0.037294686 |
| *Z0720* | hypothetical protein | | >-6.4 | | 0.0430641822 |
| *Z1034* | DNA protection during starvation conditions | | >-6.4 | | 0.018998381 |
| *Z3367* | putative exonuclease of prophage CP-933V | | >-6.3 | | 0.041137665 |
| *Z2232* | alcohol dehydrogenase | | >-6.3 | | 0.0430641822 |
| *Z4512* | hypothetical protein | | >-6.2 | | 0.0191275791 |
| *Z6031* | putative tail assembly protein of cryptic prophage CP-933P | | >-6.2 | | 0.0430641822 |
| *Z0858* | hypothetical protein | | >-6.1 | | 0.046846084 |
| *Z1969* | putative PTS system enzyme I | | >-6.1 | | 0.0282608696 |
| *Z3640* | hypothetical protein | | >-6.0 | | 0.042173913 |
| *Z5876* | putative oxidoreductase | | >-6.0 | | 0.0143888808 |
| *Z3313* | putative tail component of prophage CP-933V | | >-6.0 | | 0.042561569 |
| *Z1923* | unknown protein encoded by prophage CP-933X | | >-5.9 | | 0.028556984 |
| *Z2695* | hypothetical protein | | >-5.9 | | 0.018998381 |
| *Z4932* | cytoplasmic trehalase | | >-5.8 | | 0.0187025842 |
| *Z4922* | hypothetical protein | | >-5.7 | | 0.047232635 |
| *Z3895* | hypothetical protein | | >-5.7 | | 0.048188339 |
| *Z0480* | induced by phosphate starvation | | >-5.6 | | 0.048524039 |
| *Z5756* | outer membrane lipoprotein (lipocalin) | | >-5.6 | | 0.008459181 |
| *Z1008* | putative synthetase | | >-5.5 | | 0.0109379181 |
| *Z5709* | hypothetical protein | | >-5.5 | | 0.008163923 |
| *Z2658* | hypothetical protein | | >-5.4 | | 0.049529827 |
| *Z3045* | hypothetical protein | | >-5.4 | | 0.010820164 |
| *Z1818* | putative antirepressor protein encoded by prophage CP-933N | | >-5.3 | | 0.0143888808 |
| *Z3363* | putative single-stranded DNA binding protein of prophage CP-933V | | >-5.3 | | 0.024430933 |
| *Z1971* | putative dihydroxyacetone kinase | | >-5.2 | | 0.036252813 |
| *Z3959* | succinate-semialdehyde dehydrogenase, NADP-dependent activity | | >-5.1 | | 0.028732664 |
| *Z4930* | glutamate decarboxylase isozyme | | >-5.1 | | 0.0282608696 |
| *Z5728* | hypothetical protein | | >-5.0 | | 0.0266666667 |
| *Z4722* | induced in stationary phase, recognized by rpoS, affects cell division | | >-5.0 | | 0.048524039 |
| *Z2824* | hypothetical protein | | >-4.9 | | 0.007346457 |
| *Z3721* | transketolase | | >-4.9 | | 0.004410865 |
| *Z1873* | endodeoxyribonuclease RUS (Holliday junction resolvase) of prophage CP-933X | | >-4.8 | | 0.013787392 |
| *Z2215* | glutamate decarboxylase isozyme | | >-4.8 | | 0.003578458 |
| *Z1440* | putative single-stranded DNA binding protein | | >-4.7 | | 0.002216564 |
| *Z2837* | hypothetical protein | | >-4.7 | | 0.004283897 |
| *Z1970* | putative dihydroxyacetone kinase | | >-4.6 | | 0.044029992 |
| *Z1449* | putative regulatory protein CII of bacteriophage BP-933W | | >-4.6 | | 0.0186956522 |
| *Z2269* | hypothetical protein | | >-4.5 | | 0.049975819 |
| *Z3357* | putative regulatory protein CII of prophage CP-933V | | >-4.5 | | 0.042973421 |
| *Z4854* | acyl carrier protein | | >-4.4 | | 0.0430641822 |
| *Z2077* | unknown protein encoded by prophage CP-933O | | >-4.3 | | 0.044029992 |
| *Z2216* | acid sensitivity protein, putative transporter | | >-4.3 | | 0.049975819 |
| *Z4257* | putative coenzyme A transferase | | >-4.2 | | 0.042973421 |
| *Z4920* | putative transport ATPase | | >-4.2 | | 0.043496961 |
| *Z5978* | hypothetical protein | | >-4.1 | | 0.015185555 |
| *Z3252* | hypothetical protein | | >-4.0 | | 0.044029992 |
| *Z1435* | putative exonuclease of bacteriophage BP-933W | | >-4.0 | | 0.012795416 |
| *Z0960* | putative lysozyme protein R of prophage CP-933K | | >-3.9 | | 0.001676235 |
| *Z3079* | putative tail fiber component I of prophage CP-933U | | >-3.9 | | 0.0282608696 |
| *Z5977* | hyperosmotically inducible periplasmic protein | | >-3.8 | | 0.014225315 |
| *Z4921* | hypothetical protein | | >-3.8 | | 0.043632861 |
| *Z3249* | hypothetical protein | | >-3.7 | | 0.000673527 |
| *Z5794* | putative acyl-coenzyme A dehydrogenase | | >-3.7 | | 0.007066228 |
| *Z4890* | hypothetical protein | | >-3.7 | | 0.001313772 |
| *Z1800* | unknown protein encoded by prophage CP-933N | | >-3.6 | | 0.038199172 |
| *Z4598* | hypothetical protein | | >-3.6 | | 0.00205304 |
| *Z3720* | transaldolase | | >-3.5 | | 0.013787392 |
| *Z1439* | putative Kil protein of bacteriphage BP-933W | | >-3.5 | | 0.043632861 |
| *Z0976* | putative tail component of prophage CP-933K | | >-3.5 | | 0.000171768 |
| *Z3597* | putative minor fimbrial subunit | | >-3.4 | | 0.04447205 |
| *Z3526* | hypothetical protein | | >-3.4 | | 0.000605395 |
| *Z2950* | trehalose-6-phosphate phophatase, biosynthetic | | >-3.3 | | 0.0186956522 |
| *Z1960* | hypothetical protein | | >-3.3 | | 0.000755559 |
| *Z2162* | hypothetical protein | | >-3.2 | | 0.027969425 |
| *Z1009* | hypothetical protein | | >-3.2 | | 0.047771114 |
| *Z5644* | hypothetical protein | | >-3.1 | | 0.00753089 |
| *Z1105* | pyruvate dehydrogenase | | >-3.1 | | 0.0423809524 |
| *Z3117* | unknown protein encoded within prophage CP-933U | | >-3.1 | | 0.021900161 |
| *Z5694* | hypothetical protein | | >-3.1 | | 0.046521739 |
| *Z3371* | unknown protein encoded within prophage CP-933V | | >-3.0 | | 1.80E-05 |
| *Z1951* | putative sporulation protein | | >-3.0 | | 9.64E-05 |
| *Z2883* | hypothetical protein | | >-3.0 | | 0.0497905886 |
| *Z3972* | hypothetical protein | | >-3.0 | | 0.0190765849 |
| *Z4980* | hypothetical protein | | >-2.9 | | 0.0121023625 |
| *Z1243* | hypothetical protein | | >-2.9 | | 0.0258820244 |
| *Z3043* | hypothetical protein | | >-2.8 | | 0.0485507246 |
| *Z3365* | putative host-nuclease inhibitor protein Gam of prophage CP-933V | | >-2.8 | | 0.0324544005 |
| *Z1819* | putative tail component K homolog encoded by prophage CP-933N | | >-2.7 | | 0.0127112298 |
| **57 upregulated genes:** | | | | | |
| *Z6011* | Hypothetical protein | | >10.9 | | 0.0282608696 |
| *Z5904* | Hypothetical protein | | >10.7 | | 0.014225315 |
| *Z5783* | Hypothetical protein | | >10.5 | | 0.043632861 |
| *Z5431* | Hypothetical protein | | >10.3 | | 0.000673527 |
| *Z5199* | Hypothetical protein | | >10.1 | | 0.007066228 |
| *Z4953* | Hypothetical protein | | >9.9 | | 0.001313772 |
| *Z4879* | Putative phosphocarrier protein | | >9.8 | | 0.046521739 |
| *Z4325* | Hypothetical protein | | >9.6 | | 9.64E-05 |
| *Z4284* | Hypothetical protein | | >9.5 | | 0.000390825 |
| *Z2402* | Unknown protein encoded within prophage CP-933R | | >8.9 | | 1.45E-05 |
| *Z2327* | Hypothetical protein | | >8.8 | | 2.29E-08 |
| *Z2283* | Hypothetical protein | | >8.7 | | 1.17E-05 |
| *Z2175* | Hypothetical protein | | >8.6 | | 0.046521739 |
| *Z2095* | Unknown protein encoded within prophage CP-933O | | >8.5 | | 0.043632861 |
| *Z2091* | Unknown protein encoded within prophage CP-933O | | >8.4 | | 0.000673527 |
| *Z2089* | Unknown protein encoded within CP-933O | | >8.3 | | 0.007066228 |
| *Z1826* | Putative IS encoded protein | | >8.2 | | 0.001313772 |
| *Z1805* | Unknown protein encoded by prophage CP-933N | | >8.1 | | 0.038199172 |
| *Z1649* | Hypothetical protein | | >7.9 | | 0.00205304 |
| *Z1647* | Partial transposase | | >7.8 | | 0.013787392 |
| *Z1619* | Hypothetical protein | | >7.7 | | 0.043632861 |
| *Z1590* | Hypothetical protein | | >7.7 | | 0.000171768 |
| *Z1408* | Suppresses fabA and ts growth mutation | | >7.6 | | 0.0282608696 |
| *Z1372* | Unknown protein encoded by cryptic prophage CP-933M | | >7.6 | | 0.014225315 |
| *Z1366* | Unknown protein encoded by cryptic prophage CP-933M | | >7.5 | | 0.043632861 |
| *Z1361* | Unknown protein encoded by cryptic prophage CP-933M | | >7.5 | | 0.000673527 |
| *Z1352* | Putative endolysin of cryptic prophage CP-933M | | >7.4 | | 0.007066228 |
| *Z1185* | Hypothetical protein | | >7.4 | | 0.00205304 |
| *Z0664* | Hypothetical protein | | >7.3 | | 0.013787392 |
| *Z0489* | Hypothetical protein | | >7.3 | | 0.043632861 |
| *Z0336* | Putative regulatory protein encoded in prophage CP-933I | | >7.2 | | 0.000171768 |
| *Z1185* | Hypothetical protein | | >7.2 | | 0.0282608696 |
| *Z0664* | Hypothetical protein | | >7.1 | | 0.014225315 |
| *Z4104* | Hypothetical protein | | >7.0 | | 0.043632861 |
| *Z0489* | Hypothetical protein | | >7.0 | | 0.000673527 |
| *Z4007* | PTS system, glucitol/sorbitol-specific IIB component and second of two IIC components; frag | | >7.0 | | 0.007066228 |
| *Z0336* | Putative regulatory protein encoded in prophage CP-933I | | >6.9 | | 0.001313772 |
| *Z3946* | Putative DNA binding protein | | >6.7 | | 0.038199172 |
| *Z4104* | Hypothetical protein | | >6.6 | | 0.633150092 |
| *Z3941* | Hypothetical protein | | >6.6 | | 0.0315120563 |
| *Z4007* | PTS system, glucitol/sorbitol-specific IIB component and second of two IIC components; frag | | >6.6 | | 0.0438083378 |
| *Z3938* | Hypothetical protein | | >6.5 | | 0.0282110913 |
| *Z3946* | Putative DNA binding protein | | >6.4 | | 1.19E-05 |
| *Z3923* | Hypothetical protein | | >6.3 | | 0.0394643219 |
| *Z3941* | Hypothetical protein | | >6.2 | | 0.0256416111 |
| *Z3531* | Hypothetical protein | | >6.1 | | 0.0485507246 |
| *Z3938* | Hypothetical protein | | >5.9 | | 0.0497905886 |
| *Z3368* | Unknown protein encoded within prophage CP-933V | | >5.1 | | 0.0218309247 |
| *Z3923* | Hypothetical protein | | >4.8 | | 0.0215077178 |
| *Z3353* | Unknown protein encoded within prophage CP-933V | | >4.7 | | 0.0394643219 |
| *Z3531* | Hypothetical protein | | >4.6 | | 0.047920735 |
| *Z3334* | Unknown protein encoded within prophage CP-933V | | >3.5 | | 0.0208991923 |
| *Z3368* | Unknown protein encoded within prophage CP-933V | | >3.4 | | 0.0497905886 |
| *Z2975* | Unknown protein encoded by prophage CP-933T | | >2.9 | | 0.0497905886 |
| *Z3353* | Unknown protein encoded within prophage CP-933V | | >2.5 | | 0.015094725 |
| *Z2828* | Hypothetical protein | | >2.3 | | 0.0497905886 |
| *Z3334* | Unknown protein encoded within prophage CP-933V | | >2.1 | | 0.0183219482 |

a: Fold change<-2 or >2.

b: P_value_<0.05.
