# Supplementary material for: A Novel Small RNA Promotes Motility and Virulence of Enterohemorrhagic Escherichia coli O157:H7 in Response to Ammonium
Source: mBio. 2021 Mar 9;12(2):e03605-20. doi: 10.1128/mBio.03605-20 (PMC8092317; doi:10.1128/mBio.03605-20)
Supplement: TABLE S2 [file mBio.03605-20-st002.docx]

**Table S2.** Prevalence of *esrF* among different *E. coli* strains.

| **Strains** | **Pathotype or Origin** |
| --- | --- |
| *E. coli* NADC 5670 6564 | Enterohemorrhagic *E. coli* |
| *E. coli* NADC 5670 6565 | Enterohemorrhagic *E. coli* |
| *E. coli* O157 WS4202 | Enterohemorrhagic *E. coli* |
| *E. coli* O157 ELD 933-1 | Enterohemorrhagic *E. coli* |
| *E. coli* O157 ELD 933 | Enterohemorrhagic *E. coli* |
| *E. coli* O157 Sakai | Enterohemorrhagic *E. coli* |
| *E. coli* SRCC 1675 | Enterohemorrhagic *E. coli* |
| *E. coli* O157 3384 | Enterohemorrhagic *E. coli* |
| *E. coli* O157 TW14588 | Enterohemorrhagic *E. coli* |
| *E. coli* PA20 | Enterohemorrhagic *E. coli* |
| *E. coli* O157 8368 | Enterohemorrhagic *E. coli* |
| *E. coli* Xuzhou21 | Enterohemorrhagic *E. coli* |
| *E. coli* O157 SS52 | Enterohemorrhagic *E. coli* |
| *E. coli* O157 SS17 | Enterohemorrhagic *E. coli* |
| *E. coli* O157 JEONG-1266 | Enterohemorrhagic *E. coli* |
| *E. coli* O157 TW14359 | Enterohemorrhagic *E. coli* |
| *E. coli* O157 EC4115 | Enterohemorrhagic *E. coli* |
| *E. coli* 28RC1 | Enterohemorrhagic *E. coli* |
| *E. coli* O157 FRIK2533 | Enterohemorrhagic *E. coli* |
| *E. coli* O157 FRIK2069 | Enterohemorrhagic *E. coli* |
| *E. coli* O157 FRIK2455 | Enterohemorrhagic *E. coli* |
| *E. coli* O157 FRIK944 | Enterohemorrhagic *E. coli* |
| *E. coli* O157 664-PT8 | Enterohemorrhagic *E. coli* |
| *E. coli* O157 180-PT54 | Enterohemorrhagic *E. coli* |
| *E. coli* O157 2159 | Enterohemorrhagic *E. coli* |
| *E. coli* O157 2149 | Enterohemorrhagic *E. coli* |
| *E. coli* O157 1130 | Enterohemorrhagic *E. coli* |
| *E. coli* O157 9234 | Enterohemorrhagic *E. coli* |
| *E. coli* O157 4276 | Enterohemorrhagic *E. coli* |
| *E. coli* O55 CB9615 | Enteropathogenic *E. coli* |
| *E. coli* 2013C-4465 | Enteropathogenic *E. coli* |
| *E. coli* O55 RM12579 | Enteropathogenic *E. coli* |
| *E. coli* APEC-IMT5155 | Avian pathogenic *E. coli* |
| *E. coli* UTI89 | Extraintestinal pathogenic *E. coli* |
| *E. coli* RS218 | Neonatal-meningitis-associated *E. coli* |
| *E. coli* PMV-1 | Uropathogenic *E. coli* |
| *E. coli* UM146 | Enteroinvasive *E. coli* |
| *E. coli* SF-173 | Clinical isolate *E. coli* from bloodstream infections |
| *E. coli* IHE3034 | Neonatal-meningitis-associated *E. coli* |
| *E. coli* SF-088 | Clinical isolate *E. coli* from bloodstream infections |
| *E. coli* S88 | Neonatal-meningitis-associated *E. coli* |
| *E. coli* SF-166 | Clinical isolate *E. coli* from bloodstream infections |
| *E. coli* APEC O1 | Avian pathogenic *E. coli* |
| *E. coli* SF-468 | Clinical isolate *E. coli* from bloodstream infections |
| *E. coli* O127:H6 E2348-69 | Enterohemorrhagic *E. coli* |
| *E. coli* 2009C-3133 | Shiga toxin-producing *E. coli* |
| *E. coli* G749 | [Clinical isolate *E. coli*](https://www.ncbi.nlm.nih.gov/biosample/11319406) |
| *E. coli* SaT040 | Clinical isolate *E. coli* |
| *E. coli* ZH063 | [Clinical isolate *E. coli*](https://www.ncbi.nlm.nih.gov/biosample/11319406) |
| *E. coli* JJ1897 | [Clinical isolate *E. coli*](https://www.ncbi.nlm.nih.gov/biosample/11319406) |
| *E. coli* CD306 | [Clinical isolate *E. coli*](https://www.ncbi.nlm.nih.gov/biosample/11319406) |
| *E. coli* MNCRE44 | [Clinical isolate *E. coli*](https://www.ncbi.nlm.nih.gov/biosample/11319406) |
| *E. coli* O25b:H4 | Uropathogenic *E. coli* |
| *E. coli* NCTC 13441 | Uropathogenic *E. coli* |
| *E. coli* JJ1887 | [Clinical isolate *E. coli*](https://www.ncbi.nlm.nih.gov/biosample/11319406) |
| *E. coli* JJ1886 | [Clinical isolate *E. coli*](https://www.ncbi.nlm.nih.gov/biosample/11319406) |
| *E. coli* O25b:H4 ST131 EC958 | Uropathogenic *E. coli* |
| *E. coli* uk P46212 | [Clinical isolate *E. coli*](https://www.ncbi.nlm.nih.gov/biosample/11319406) |
| *E. coli* JJ2434 | [Clinical isolate *E. coli*](https://www.ncbi.nlm.nih.gov/biosample/11319406) |
| *E. coli* Ecol 732 | [Clinical isolate *E. coli*](https://www.ncbi.nlm.nih.gov/biosample/11319406) |
| *E. coli* ZH193 | [Clinical isolate *E. coli*](https://www.ncbi.nlm.nih.gov/biosample/11319406) |
| *E. coli* SE15 | [Clinical isolate *E. coli*](https://www.ncbi.nlm.nih.gov/biosample/11319406) |
| *E. coli* Ecol 743 | [Clinical isolate *E. coli*](https://www.ncbi.nlm.nih.gov/biosample/11319406) |
| *E. coli* MVAST0167 | [Clinical isolate *E. coli*](https://www.ncbi.nlm.nih.gov/biosample/11319406) |
| *E. coli* Eco889 | Environmental isolate *E. coli* |
| *E. coli* Eco745 | Environmental isolate *E. coli* |
| *E. coli* Eco448 | Environmental isolate *E. coli* |
| *E. coli* ECONIH2 | Hospital-associated carbapenemase-producing *Enterobacteriaceae* |
| *E. coli* K15KW01 | Uropathogenic *E. coli* |
| *E. coli* O83:H1 NRG 857C | Clinical isolate *E. coli* from Crohn's disease |
| *E. coli* LF82 | Avian pathogenic *E. coli* |
| *E. coli* ABU 83972 | Uropathogenic *E. coli* |
| *E. coli* cloned i2 | Uropathogenic *E. coli* |
| *E. coli* cloned i14 | Uropathogenic *E. coli* |
| *E. coli* CFT073 | Uropathogenic *E. coli* |
| *E. coli* Nissle 1917 | Clinical isolate *E. coli* from Ulcerative colitis |
| *E. coli* UPEC 26 1 | Uropathogenic *E. coli* |
| *E. coli* ECONIH1 | Hospital-associated carbapenemase-producing *Enterobacteriaceae* |
| *E. coli* MS6198 | Uropathogenic *E. coli* |
| *E. coli* ST648 | Clinical isolate *E. coli* from pleural effusion of patients with empyema thoracis |
| *E. coli* SMS-3-5 | Environmental isolate *E. coli* |
| *E. coli* O7:K1 CE10 | Neonatal-meningitis-associated *E. coli* |
| *E. coli* IAI39 | Uropathogenic *E. coli* |
