# Supplementary material for: A Novel Small RNA Promotes Motility and Virulence of Enterohemorrhagic Escherichia coli O157:H7 in Response to Ammonium
Source: mBio. 2021 Mar 9;12(2):e03605-20. doi: 10.1128/mBio.03605-20 (PMC8092317; doi:10.1128/mBio.03605-20)
Supplement: TABLE S3 [file mBio.03605-20-st003.docx]

**Table S3.** Bacterial strains, plasmids, and primers used in this study.

| **Strains/Plasmids/Primers** | | **Genotype or description** | | **Source or reference** |
| --- | --- | --- | --- | --- |
| **Strains** |  | |  | |
| WT/G2734 | Wild-type EHEC serotype O157: H7 strain EDL933 | | ATCC^a^ | |
| WT^Nal^ | WT strain induced with resistance of Nalidixic acid; Nal^R^ | | This study | |
| Δ*esrF* | sRNA coding gene *esrF* deletion mutant in WT; Cm^R^ | | This study | |
| Δ*esrF*+P*esrF* | Δ*esrF* strain containing plasmid pBlue-*esrF*; Ap^R^+Cm^R^ | | This study | |
| Δ*ntrC* | *ntrC* deletion mutant in WT; Cm^R^ | | This study | |
| Δ*ntrC*+P*ntrC* | Δ*ntrC* strain containing plasmid pBlue-*ntrC*; Ap^R^+Cm^R^ | | This study | |
| Δ*hfq* | *hfq* deletion mutant in WT; Cm^R^ | | This study | |
| Δ*hfq*+P*hfq* | Δ*hfq* strain containing plasmid pBlue-*hfq*; Ap^R^+Cm^R^ | | This study | |
| Δ*glnA*p | *glnA* promoter deletion mutant in WT; Cm^R^ | | This study | |
| Δ*flhB* | *flhB* deletion mutant in WT; Cm^R^ | | This study | |
| Δ*flhB*+P*flhB* | Δ*flhB* strain containing plasmid pBlue-*flhB*; Ap^R^+Cm^R^ | | This study | |
| Δ*esrF*Δ*flhB* | Δ*esrF* and Δ*flhB* deletion mutant in WT;Cm^R^+Kan^R^ | | This study | |
| BL21 pET28a-*ntrC*-His | BL21 strain containing plasmid pET28a-*ntrC*-His; Kan^R^ | | This study | |
| WT-*rfp* | WT strain containing constitutively expressed *rfp* gene; Cm^R^ | | This study | |
| Δ*ntrC*-*rfp* | Δ*ntrC* strain containing constitutively expressed *rfp* gene; Cm^R^+Kan^R^ | | This study | |

| **Plasmids** |  |  |
| --- | --- | --- |
| pKD46 | Red recombinase system under an Arabinose-inducible promoter; Ap^R^ | Lab collection |
| pKD3 | Template plasmid containing the Cm cassette for λ Red recombination; Cm^R^ | Lab collection |
| pKD4 | Template plasmid containing the Km cassette for λ Red recombination; Kan^R^ | Lab collection |
| pJV300 | Promoter-less plasmid to express sRNA; Ap^R^ | Yanjie Chao,2012 |
| pJV300-*esrF* | Promoter-less plasmid used in Fig 1A with *esrF* promoter and backbone; Ap^R^ | This study |
| pET28a+ | T7 expression vector; Kan^R^ | Lab collection |
| pET28a+-*ntrC* | pET28a carrying *ntrC* tagged with 6*His tag; Kan^R^ | This study |
| pBluesript-II-SK- (+) | Complement vector; Ap^R^ | Lab collection |
| pBlue-*esrF* | pBluesript -II-SK- (+) carrying EsrF coding sequence and its promoter region; Ap^R^ | This study |
| pBlue-*flhB* | pBluesript -II-SK- (+) carrying *flhB* and its promoter region; Ap^R^ | This study |
| pBlue-*ntrC* | pBluesript -II-SK- (+) carrying *ntrC* and its promoter region; Ap^R^ | This study |
| pBlue-*hfq* | pBluesript -II-SK- (+) carrying *hfq* and its promoter region; Ap^R^ | This study |
| pETDuet-1 | Expression vector, Ap^R^ | Lab collection |
| pETDuet-*esrF*p-*gfp* | pETDuet-1 carrying  *esrF* promoter fused *gfp*, Ap^R^ | This study |
| pETDuet-*flhB*p-*gfp* | pETDuet-1 carrying  *flhB* promoter fused *gfp*, Ap^R^ | This study |
| pETDuet-*ntrC*p-*gfp* | pETDuet-1 carrying  *ntrC* promoter fused *gfp*, Ap^R^ | This study |
| **Primers** | **Sequence (5’→3’)** | **Function** |
| *esrF*-mut-F | TTTACGGATAAAAAGCGCGAAGCATCAGAGAATTGACGGGTGTAGGCTGGAGCTGCTTC | *esrF* deletion |
| *esrF*-mut-R | CCGCATCCGGTAGAGTTTGAGCTGTACTACAGCGTCTAAATGGGAATTAGCCATGGTCC | *esrF* deletion |
| *ntrC*-mut-F | atgcaacgagggatagtctgggtagtcgatgacgatagtGTGTAGGCTGGAGCTGCTTC | *ntrC* deletion |
| *ntrC*-mut-R | tcactccatccccagctcttttaacttacgcgtcagggtATGGGAATTAGCCATGGTCC | *ntrC* deletion |
| *hfq*-mut-F | AGGTTCAAAGTACAAATAAGCATATAAGGAAAAGAGAGAGTGTAGGCTGGAGCTGCTTC | *hfq* deletion |
| *hfq*-mut-R | GATCGCTGGCTCCCCGTGTAAAAAAAACAGCCCGAAACCATGGGAATTAGCCATGGTCC | *hfq* deletion |
| *flhB*-mut-F | ttactcatgggtcggtttctcgttaataaaatccagggcttccggcaccggaagatgagGTGTAGGCTGGAGCTGCTTCG | *flhB* deletion |
| *flhB*-mut-R | gtgtctgacgagagcgacgacaaaacagaagcccccacacctcaccgactagaaaaagcCATATGAATATCCTCCTTAG | *flhB* deletion |
| *ntrC*-28a-His-F | CATGCCATGGGCATGCAACGAGG | *ntrC* cloning |
| *ntrC*-28a-His-R | CCGCTCGAGCTCCATCCCCAGCTC | *ntrC* cloning |
| *esrF*-pblue-F | CCCAAGCTTcggatgaagcgatcgatgcgt | *esrF* cloning |
| *esrF*-pblue-R | CCGCTCGAGaagcatcagagaattgacggag | *esrF* cloning |
| *flhB*-pblue-F | CCCAAGCTTaaaagccTtaaatcccgcctg | *flhB* cloning |
| *flhB*-pblue-R | CCGCTCGAGttactcatgggtcgg | *flhB* cloning |
| *ntrC*-pblue-F | CCCAAGCTTggaagataacgggccaggcattcc | *ntrC* cloning |
| *ntrC*-pblue-R | CCGCTCGAGtcactccatccccagctctt | *ntrC* cloning |
| EsrF-sense-pGEM-F | CATGCCATGGtactacagcgtctaatagttgaagt | EsrF+ cloning |
| EsrF-sense-pGEM-R | TCCCCCCGGGGcgaagcatcagagaattgacggag | EsrF+ cloning |
| EsrF-reverse-pGEM-F | CATGCCATGGgcgaagcatcagagaattgacggag | EsrF- cloning |
| EsrF-reverse-pGEM-R | TCCCCCCGGGtactacagcgtctaatagttgaagt | EsrF- cloning |
| *flhB*-mRNA-pGEM-F | CATGCCATGGgccaaaccagataacactaacgcc | *flhB* cloning |
| *flhB*-mRNA-pGEM-R | TCCCCCCGGGtgaccacgtcatatcaggcggtct | *flhB* cloning |
| *rpoA*-RT-F | cgcgctgcttcaacattgtaggca | RT-PCR |
| *rpoA*-RT-R | tatcaaagttcagcgcggtcgtgg | RT-PCR |
| EsrF-RT-F | cgcgaagcatcagagaattgacgg | RT-PCR |
| EsrF-RT-R | cgcatccggtagagtttgagctgt | RT-PCR |
| *flhB*-RT-F | tttaacatgggggtcgccttc | RT-PCR |
| *flhB*-RT-R | atgcgatggatttggtagggc | RT-PCR |
| *fliA*-RT-F | tcttcgcgccactcatcgtaggag | RT-PCR |
| *fliA*-RT-R | ggcacaggcaatagggcaactgg | RT-PCR |
| *fliC*-RT-F | aattgaagatgtcgcggcagccgc | RT-PCR |
| *fliC*-RT-R | acgctggtagcgcagctaaagctg | RT-PCR |
| *ntrC*-RT-F | Cgagaaaggcgcgtttaccggcgc | RT-PCR |
| *ntrC*-RT-R | ccgcacatccactttcaccggcgc | RT-PCR |
| 5S rRNA RNA probe | DIG-5’-cacacuaccaucggcgcuacggcguuucacuucugaguucggcaugggguc | Northern blotting |
| EsrF RNA probe | DIG-5’-UgggcUgaaagUUUccacggcaacUaaaUcccggcgUUgUUgcgccggg | Northern blotting |
| *flhB* mRNA probe | DIG-5’- gUgcgcaUagcccUaccaaaUccaUcgcaUUacccaUggcggUaaUcggag | Northern blotting |
| EsrF-5’RACE-F1 | ctaaatcccggcgttgttgcg | RACE |
| EsrF-5’RACE-F2 | GTTGTTGCGCCGGGTAGTACA | RACE |
| EsrF-5’RACE-R1 | GCCGTGGAAACTTTCAGCCCA | RACE |
| EsrF-5’RACE-R2 | TCAGCCCATCTCTGCATGGGC | RACE |
| EsrF-3’RACE-F-GSP | GAAACTTTCAGCCCAT | RACE |
| EsrF-3’RACE-R-GSP | CTGAAAGTTTCCACGGCA | RACE |
| EsrF-3’RACE-R-nested-GSP | TAAATCCCGGCGTTGTTGCGC | RACE |
| P_EsrF_-F | ggctgaaagtttccacggcaactaa | protein-DNA EMSA |
| P_EsrF_-R | aagaagcactgaacgagctggatc | protein-DNA EMSA |
| P_Kan_-F | GCACTCAGGGCGCAAGGGCTGCTAAAGG | protein-DNA EMSA |
| P_Kan_-R | TTGCAGGGCTTCCCAACCTTACCAG | protein-DNA EMSA |
| P_EsrF_mut_-F2 | gcgctagacgtactgactgagaattgacggagaaaaaagccc | protein-DNA EMSA |
| P_EsrF_mut_-R2 | agtcagtacgtctagcgcatccgtaaaaagctataatgcact | protein-DNA EMSA |
| EsrF-*in vitro*-F | TAATACGACTCACTATAGGGaggttgcaggctctctggaagaagcactgaacgagctggatctggacc | *In vitro* transcription |
| EsrF-*in vitro*-R | AAACAAACAGATAAAAGAACAGATGAAAGAACAGATAAGAAAACAGATGGAAgcccatgcagaratgggctgaaag | *In vitro* transcription |
| EsrF-complement-*in vitro* -F | TAATACGACTCACTATAGGGgggctgaaagtttccacggcaactaaatcccggcgttgttgcgccggg | *In vitro* transcription |
| EsrF- complement-*in vitro*-R | AAACAAACAGATAAAAGAACAGATGAAAGAACAGATAAGAAAACAGATGGAAaggttgcaggctctctggaagaag | *In vitro* transcription *In vitro* transcription |
| *flhB*-mRNA-*in vitro*-F | TAATACGACTCACTATAGGGtgaccacgtcatatcaggcggtctgataaggcgatgacgccgcatccga | *In vitro* transcription |
| *flhB*-mRNA-*in vitro*-R | AAACAAACAGATAAAAGAACAGATGAAAGAACAGATAAGAAAACAGATGGAAgccaaaccagataacactaacgcc | *In vitro* transcription |

^a^ ATCC: American Type Culture Collection, Manassas, Virginia, USA.
